# NF-κB-dependent GR cistrome redistribution recruits GR to inflammatory genes but correlates with lesser glucocorticoid-mediated repression

**DOI:** 10.1016/j.isci.2025.114206

**Published:** 2025-11-25

**Authors:** Mahmoud M. Mostafa, Amandah Necker-Brown, Alex Gao, Andrew J. Thorne, Akanksha Bansal, Lucy Swift, Annika M. Maj, Sarah K. Sasse, Pina Colarusso, Anthony N. Gerber, Robert Newton

**Affiliations:** 1 Lung Health Research Group, Snyder Institute for Chronic Diseases, Cumming School of Medicine, University of Calgary, Calgary, AB, Canada; 2 Department of Physiology and Pharmacology, Cumming School of Medicine, University of Calgary, Calgary, AB, Canada; 3 Department of Medicine, National Jewish Health, Denver, CO, USA; 4 Department of Immunology and Genomic Medicine, National Jewish Health, Denver, CO, USA; 5 Department of Medicine, University of Colorado, Aurora, CO, USA

**Keywords:** Genomics, Gene network, Molecular mechanism of gene regulation

## Abstract

While ligand-activated glucocorticoid receptor (GR) binds DNA to activate transcription, glucocorticoids, including budesonide, reduce inflammatory gene expression, yet recruit GR to many such gene loci. In epithelial cells, the inflammatory cytokine, interleukin-1β (IL1B), activates nuclear factor (NF)-κB to induce gene expression, and co-treatment with budesonide produces nanoscale GR-RELA nuclear co-localization. Such co-stimulation orchestrated reciprocal genome-wide redistribution of GR- and RELA-binding regions (GBRs and RBRs, respectively) relative to each mono-treatment to produce widespread GBR-RBR overlap. This correlated with increased RNA polymerase-2 presence and required NF-κB for GR cistrome remodeling. Mapping transcription start sites to the nearest GBR or RBR each revealed associations with upregulated, but not repressed, genes. Importantly, RBR proximity to budesonide-upregulated genes and GBR proximity to IL1B-upregulated genes correlated with attenuated repression on co-treatment. As this occurred on a background of glucocorticoid-induced repression, GR presence at specific IL1B-induced gene loci may reduce/protect from an otherwise more prevalent glucocorticoid-induced repression.

## Highlights

• GR and RELA recruitment correlate with RNA polymerase-2 and gene upregulation
• IL1B-plus-budesonide redistributes each cistrome to produce mutual GR/RELA overlap
• GR cistrome redistribution is NF-κB dependent
• Inflammatory genes that recruit GR reveal less repression by budesonide

## Graphical abstract

**Figure:**
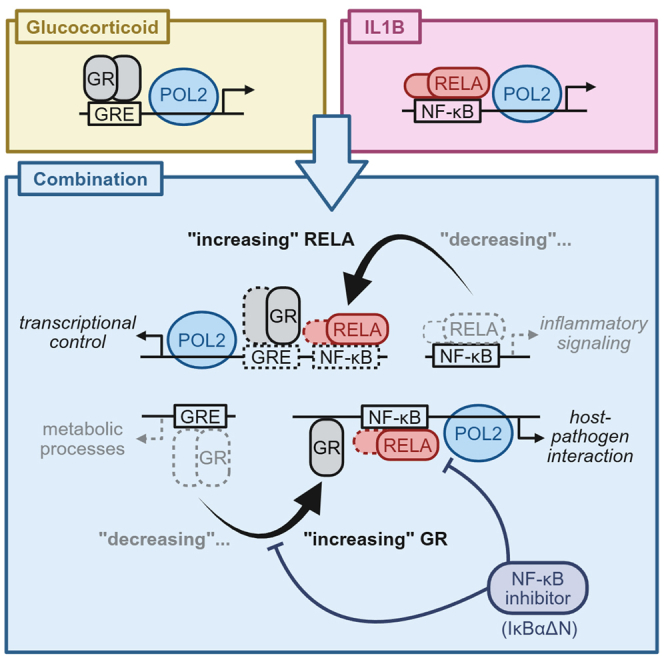

## Introduction

Acting via the glucocorticoid receptor ([GR] *NR3C1*), glucocorticoids, including dexamethasone and budesonide, attenuate inflammatory gene expression and provide benefit in inflammatory diseases.^1^^,^^2^ GR agonists promote nuclear translocation to elicit *trans* effects via *cis*-acting palindromic DNA motifs, or glucocorticoid response elements (GREs), where GR recruits coactivators to activate transcription. Such “transactivation” mechanisms were once thought mainly responsible for the side effects of glucocorticoid therapy, whereas repression of inflammatory gene expression was widely attributed to GR acting in *trans* at target loci to repress gene transcription (“transrepression”).^1^^,^^2^

Conversely, an increased appreciation for GR transactivation in driving indirect mechanisms of repression now exists.^3^^,^^4^ Thus, glucocorticoids increase expression of many genes that target inflammation. These include *NFKBIA*, encoding inhibitor of κBα (IκBα), an endogenous inhibitor of nuclear factor (NF)-κB,^5^^,^^6^ a key inflammatory transcription factor that is made up of p50 and the main transactivating subunit, RELA.^7^ Other examples include *DUSP1*, a phosphatase that inactivates mitogen-activated protein kinases (MAPKs)^8^^,^^9^; *TNFAIP3*, a combined deubiquitinase/ubiquitin ligase that terminates signaling to NF-κB^10^; and a dominant-negative interleukin (IL) 1 receptor-associated kinase (IRAK), *IRAK3*, which reduces toll-like receptor signaling.^11^ Indeed, RNA and chromatin immunoprecipitation (ChIP) sequencing (RNA-seq and ChIP-seq, respectively) supports GR-dependent activation of these genes.^12^^,^^13^^,^^14^^,^^15^^,^^16^ Further, these “anti-inflammatory” genes are not merely transactivated by GR but are independently upregulated by inflammatory stimuli to control inflammation.^17^ At these loci, GR-binding regions (GBRs) occur near RELA-binding regions (RBRs) where GR and NF-κB/RELA may cooperate to enhance transcription.^17^ Such findings that GR-dependent transactivation is critical for repression also align with the numerous reports that glucocorticoid-induced repression of gene expression is blocked by transcriptional and/or translational inhibition.^3^^,^^18^^,^^19^ Furthermore, post-transcriptional and translational mechanisms of repression are mechanistically distinct from direct GR transrepression.^19^ Thus, mRNA stabilization and translation involving MAPKs are reduced by GR-dependent transactivation of *DUSP1*,^20^ which will act to suppress MAPK signaling. While this highlights regulatory genes that are upregulated by both inflammatory stimuli and glucocorticoids, genes including *TSC22D3*, which repress activator protein 1 (AP-1) and NF-κB activity, are solely glucocorticoid induced and represent another distinct mode of glucocorticoid-dependent repression.^21^

Notwithstanding the above, GR recruitment to gene loci is also proposed to directly transrepress transcription from the so-called negative GREs.^1^^,^^22^ Examples include GR binding at inverted repeats or tethering via NF-κB or AP-1 to enable repressor recruitment.^23^^,^^24^^,^^25^ Since, with an inflammatory stimulus plus glucocorticoid, GR recruits to AP-1 and NF-κB-binding regions at both anti- and pro-inflammatory gene loci,^13^^,^^14^^,^^15^^,^^16^^,^^26^ uncertainty now exists concerning the functional role of GR. To explore this question, pulmonary A549 epithelial cells, an adenocarcinoma line,^27^ were used to explore relationships between activated GR and NF-κB/RELA and relate this to changes in regulated gene expression. In these cells, IL-1β (IL1B)-induced NF-κB activation occurs rapidly and involves RELA.^28^^,^^29^ This upregulates NF-κB-dependent gene expression, which, with many downstream genes being repressed by glucocorticoids, including in primary airway epithelial cells, indicates model appropriateness.^30^^,^^31^ Budesonide promoted GBRs and IL1B generated RBRs that each correlated with RNA polymerase-2 (RNAP2) presence and associated with upregulated genes. IL1B-plus-budesonide co-treatment redistributed GR to RBRs and RELA to GBRs, effects that correlated positively with RNAP2 presence and lesser repression of associated genes. Finally, despite GR redistribution requiring NF-κB, the lack of evidence for direct transrepression demands re-evaluation of roles for GR, or RELA, when recruited to binding regions of the other.

## Results

### Reciprocal loss of budesonide- and IL1B-induced transcription

In A549 cells, budesonide- and dexamethasone-driven GRE reporter activity requires GR,^30^^,^^32^^,^^33^ plus was modestly reduced by IL1B co-treatment (Figure S1A).^34^ As shown using actinomycin D to inhibit RNAP2, this GRE reporter activity primarily involved transcription 0.5–2 h post-glucocorticoid (Figure S1B), while GR expression was unaffected by IL1B (Figures S1C and S1D). Similarly, NF-κB reporter activity requires RELA and was modestly reduced on co-treatment with maximally effective concentrations of dexamethasone (1 μM) (Figure S1E)^34^ or budesonide (300 nM) (Figure S1F).^35^ This response primarily required transcription 0.5–2 h post-IL1B (Figure S1G), and neither budesonide (1 h) nor dexamethasone (1–24 h) affected RELA expression (Figures S1C and S1H). Thus, both glucocorticoid-induced GRE-dependent transcription and IL1B-induced NF-κB-dependent transcription occurred over similar 0.5- to 2-h time frames, at which expressions of both GR and RELA were unaffected by IL1B-plus-budesonide, and this establishes 1 h as a suitable time to further examine activation of each factor.

### Independent GR and RELA translocation

**Figure 1:**
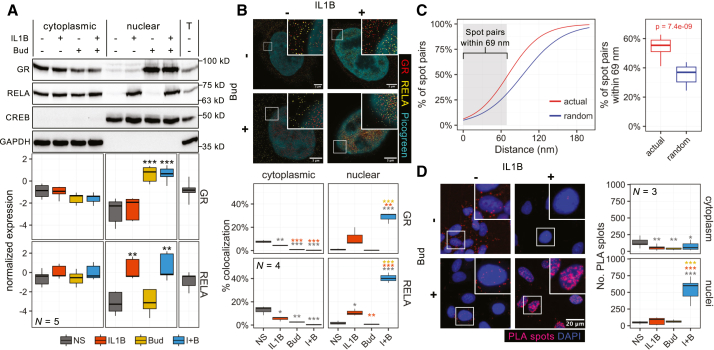
Budesonide-plus-IL1B co-treatment promotes nanoscale GR and RELA nuclear co-localization A549 cells were either not stimulated (NS), or treated with budesonide (Bud; 300 nM), IL1B (1 ng/mL), or their combination (I + B) for 1 h. (A) Cytoplasmic and nuclear lysates were blotted for GR, RELA, and GAPDH. T = total cell lysate. *N* = 4 experiments. (B) Super-resolution microscopy with STED visualized GR (red) and RELA (yellow) in the nucleus (green) and cytoplasm. Number of spots co-localizing by ≥ 1 pixel is plotted. *N* = 4 experiments, and 3–4 images were analyzed for each. Scale bars, 3 μm. (C) Center-center distances for GR-RELA spot-pairs are plotted as percentage of total for the actual data and 10 random assignments of the same spots. (D) PLA for GR and RELA (red). Number of spots in the nuclei (blue) and cytoplasm are plotted. *N* = 3 experiments, and 3–4 images were analyzed for each. Scale bar, 20 μm. Data are presented as box-and-whiskers plots. (A, B, and D) Data were analyzed by one-way ANOVA with Tukey’s post hoc test, where ∗, ∗∗, and ∗∗∗ represent *p* ≤ 0.05, *p* ≤ 0.01, and *p* ≤ 0.001, respectively. Color coding in figures indicates the comparison groups. (C) Data analyzed using Wilcoxon test and numeric *p* value is depicted.

Nuclear translocation of each factor was tested by cellular fractionation 1 h post-treatments. Budesonide reduced cytoplasmic GR and increased nuclear GR, while IL1B alone, or as co-treatment, did not affect GR translocation (Figure 1A). Similarly, IL1B induced robust nuclear accumulation of RELA at 1 h, and this was unaffected by budesonide (Figure 1A). Immunofluorescence microscopy confirmed these effects to reveal GR and RELA localization to the nucleus with co-treatment (Figure S2).

### Nanoscale GR/RELA co-localization

To test the relative proximity of GR and RELA, cells were treated for 1 h with budesonide and/or IL1B prior to super-resolution microscopy using stimulated-emission depletion (STED). This revealed punctate staining for both GR and RELA such that, with IL1B-plus-budesonide, ∼30% of GR and ∼40% of RELA nuclear puncta showed ≥1 pixel (∼20 nm) overlap with the other factor (Figure 1B). Thus, nuclear GR/RELA co-localization was significantly enhanced, with reduced cytoplasmic co-localization, with co-treatment (Figure 1B). In nuclei, comparison between spot coordinates and equivalent random assignments confirmed that GR and RELA were closer to each other than predicted by chance (Figure 1C). Further, with an average edge-to-edge distance minus center-to-center distance of 69 nm between fluorophore signals, the 54% of spot-pairs within this distance were considered touching.

Proximity ligation assay (PLA) produces signal when complementary oligonucleotide-antibody pairs are within 30–40 nm.^36^^,^^37^ Thus, probing for RELA and IκBα produced cytoplasmic PLA signals in untreated cells that disappeared after 15 min of IL1B (Figure S3A), presumably due to IκBα loss.^38^ Conversely, untreated cells showed low nuclear PLA signals between GR and the coactivator p300 that were dramatically increased by budesonide (Figure S3B), consistent with GR translocation and interaction with p300.^39^ With GR and RELA, low extra-nuclear PLA signal in untreated cells was reduced by all treatments (Figure 1D). Conversely, the low nuclear signals in untreated cells were unaffected by budesonide or IL1B, but were dramatically enhanced with IL1B-plus-budesonide. However, 5–10 nm diameters are variously indicated for 43- to 200-kDa globular proteins,^40^ distances that crystallographic data (Protein DataBank; www.rcsb.org), combined with modeling of structures from AlphaFold (AlphaFold.com), also support for GR and RELA/NF-κB (data not shown). Thus, the 30- to 40- or 69-nm overall (probe-to-probe) resolution limits from the PLA and STED data, rather than necessarily implying GR-RELA interaction, reveal a non-random closeness that then raises questions as to biological significance(s).

### Reciprocal co-modulation of RELA and GR cistromes

**Figure 2:**
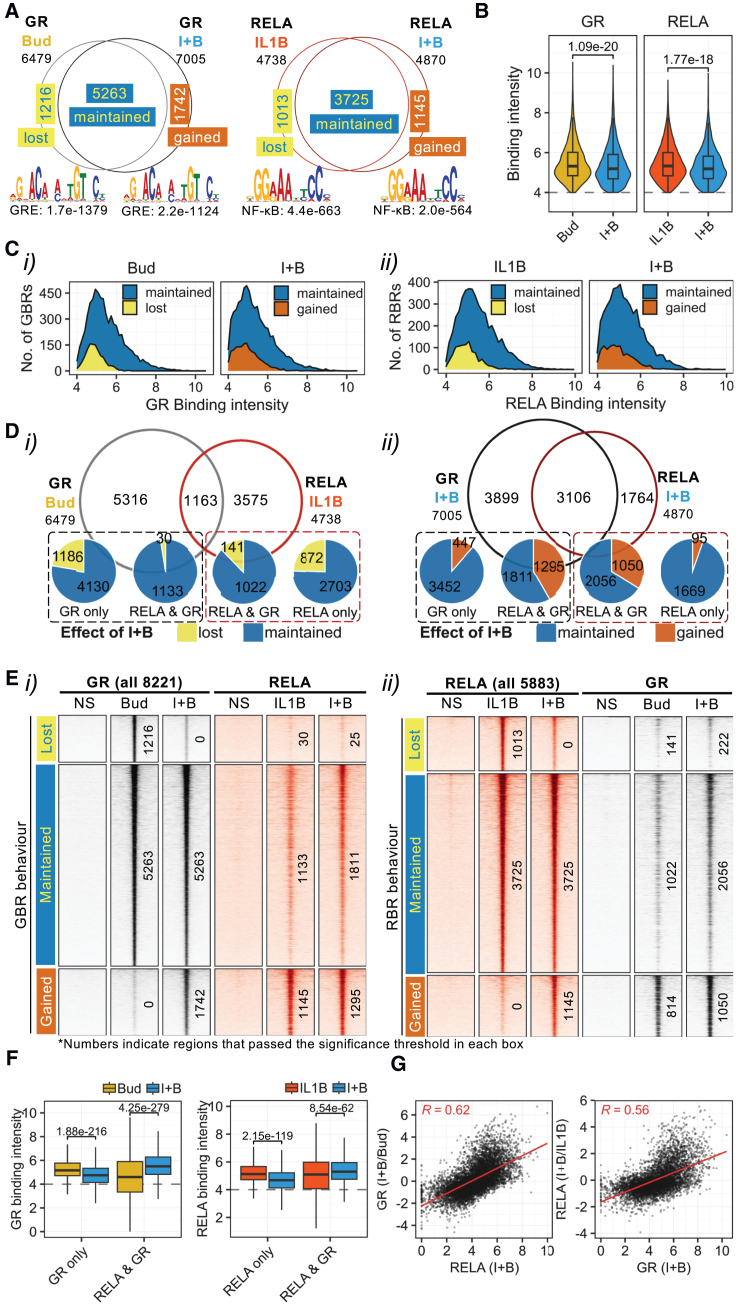
Mutual GBR and RBR loss and gain A549 cells were treated as in Figure 1 for 1 h prior to GR and RELA ChIP-seq. *N* = 2 experiments. (A) GBR and RBR number and the most enriched motifs are shown for each treatment. (B) Binding intensity (log_2_ normalized read counts) of all GBRs or RBRs with treatments. Data are presented as violin plot with impeded box-and-whiskers plots and were analyzed using Mann-Whitney U test; numeric *p* values are depicted. (C) Binding intensity profiles for (i) GBRs and (ii) RBRs showing loss or gain on co-treatment. (D) GBR and RBR overlap with (i) mono-treatments or (ii) co-treatment. Pie charts show GBR/RBR loss or gain on co-treatment for each group. (E) Heatmaps for all (i) GBRs or (ii) RBRs centered (±1.5 kb) around summit, showing GR (black) and RELA (red) binding for each region with treatment. GBR or RBR number meeting threshold criteria is indicated. (F) Effect of treatment on GR or RELA binding intensity at non-overlapping (GR or RELA only) and overlapping (RELA & GR) regions. Data are presented as box-and-whiskers plots and were analyzed using Wilcoxon test; numeric *p* values are depicted. (G) Relationship between change in binding intensity for GR (left) or RELA (right) on co-treatment with binding intensity for the other factor at the same region on co-treatment.

GR and RELA ChIP was next used to assess the relationship between GR and RELA recruitment to DNA. DNA from GR and RELA ChIP obtained 1 h after IL1B and/or budesonide was qPCR validated (Figure S4).^14^^,^^41^ Paired-end sequencing identified 6,479 budesonide-induced GBRs and 7,005 GBRs with budesonide-plus-IL1B, giving 8,221 GBRs overall (Figure 2A). IL1B produced 4,738 RBRs and IL1B-plus-budesonide produced 4,870 RBRs to give 5,883 RBRs (Figure 2A). GBR and RBR positions relative to expressed genes revealed >50% of sites within 30 kb of transcription start sites ([TSSs] not shown), and comparisons with prior studies confirmed data quality.^12^^,^^13^^,^^14^^,^^15^^,^^16^ Robust budesonide-induced GBRs occurred at glucocorticoid-induced genes (*KLF9* and *PER1*), including regulatory genes (*DUSP1*, *NFKBIA*, *TNFAIP3*, and *ZFP36*), while IL1B recruited RELA to regulatory (*NFKBIA*, *TNFAIP3*, and *ZFP36*) and inflammatory (*LTB*-*TNF* and *BDKRB1*) gene loci (Figure S5).

Despite IL1B-plus-budesonide marginally increasing GBR number, GR binding intensity was modestly reduced (Figure 2B). Thus, with IL1B-plus-budesonide, 1,216 budesonide-induced GBRs moved below the threshold and were “lost.” Conversely, 1,742 new GBRs were “gained” and the 5,263 GBRs present with budesonide and budesonide-plus-IL1B treatments were described as “maintained” (Figures 2A and S6A). Since many higher-intensity GBRs were lost or gained, these effects were not variability at the cusp of detection but rather reflected region-specific changes (Figure 2Ci). Comparing IL1B-induced and IL1B-plus-budesonide-induced RBRs revealed 1,013 lost and 1,145 gained RBRs with overall binding intensity modestly reduced with co-treatment (Figures 2A and 2B). These effects were apparent at both low- and high-intensity RBRs, again suggesting region-specific changes (Figures 2Cii and S6B).

### Motif enrichment suggests GR/NF-κB crosstalk

To explore the nature of GR or RELA binding, motif enrichment, using MEME-ChIP,^42^ was applied to these GBRs and RBRs. This showed simple GREs as most enriched within budesonide-induced GBRs, with FOXA, SOX, CEBP, and AP-1 transcription factor families being other top motifs (Figures 2A and S7A). With IL1B-plus-budesonide GBRs, GREs again ranked first, but other motifs were displaced by NF-κB, which now ranked second (*E*-value, 1.3 × 10^−116^ from 2.4 × 10^−5^) (Figures 2A and S7B). With IL1B-induced RBRs, NF-κB motifs were most highly enriched, with CEBP, TEAD, and AP-1 families also highly enriched (Figures 2A and S7C). However, while NF-κB motifs remained most enriched with IL1B-plus-budesonide-induced RBRs, GREs now ranked second (*E*-value 1.3 × 10^−148^ from 7.1 × 10^−10^) (Figures 2A and S7D). Thus with the IL1B-plus-budesonide co-treatment, NF-κB motifs became markedly more enriched in the GR cistrome and GRE motifs became more enriched in the RELA cistrome. This supports concepts of biological interaction and possible co-regulatory effects.

### RBR/GBR overlap with IL1B-plus-budesonide

The central location of GREs and NF-κB motifs within GBRs and RBRs suggested that RELA and GR may co-localize to shared genomic regions with IL1B-plus-budesonide (Figure S7). Indeed, the 6,479 budesonide-induced GBRs and the 4,738 IL1B-induced RBRs showed 1,163 regions in common (Figure 2Di). However, with IL1B-plus-budesonide, the 7,005 GBRs and 4,870 RBRs revealed 3,106 overlapping regions, such that 63.8% of RBRs co-bound GR and 44.4% of GBRs recruited RELA (Figure 2Dii). Thus, GBRs near glucocorticoid-induced genes, such as *FKBP5*, *MT2A*, *SCL19A2*, *ZFAND5*, and *BIRC3*,^43^ showed enhanced RELA recruitment with co-treatment (Figure S8A). Similarly, IL1B-induced RBRs near IL1B-upregulated genes showed increased GR recruitment with IL1B-plus-budesonide, an effect seemingly independent of whether IL1B-induced mRNA expression was ultimately repressed (*CXCL8*, and *ICAM1*) or not (*C3*, *IL32*, and *SOD2*), with IL1B-plus-budesonide (Figure S8B). Furthermore, the regulatory genes (*DUSP1*, *NFKBIA*, *TNFAIP3*, and *ZFP36*) all revealed IL1B- and glucocorticoid-inducible mRNA expression and the presence of budesonide-induced GBRs and IL1B-induced RBRs that aligned with co-treatment (Figure S5B).

To explore distances between the peaks of GR and RELA binding, summit positions were extracted and the distance between summits for GBRs, or RBRs, were mapped to the nearest summit of the other factor in the two mono-treatments or within the IL1B-plus-budesonide co-treatment (Figures S9A and S9B). In each case, biphasic cumulative distribution plots revealed a preferential clustering of peak summits that occurred over short (<400 bp) distances followed by more widely dispersed pairings over distances up to ∼1 Mb, at which distance essentially all peak summits were paired. For both GR mapping to RELA summits and RELA mapping to GR summits, IL1B-plus-budesonide considerably increased the overall faction of short-range (≤400 bp) pairings. In addition, the median separation for both the short range and the more widely dispersed summits was reduced in the co-treatment (Figures S9C and S9D). Furthermore, distribution of the short-range pairings revealed that while summits in the two mono-treatments showed a modal average of 56 bp, this reduced to 32 bp with IL1B-plus-budesonide (Figure S9E). Taking 0.33 nm/bp,^44^ this corresponds to a simple linear distance of ∼10.5 nm and is compatible with possible physical contact between the two factors. Conversely, summit-pairs in the 32- to 400-bp interval are within, or at, the resolution limits of the PLA and STED analyses, but may not involve direct GR-RELA interaction.

### GR and RELA co-binding correlates with region behavior

Given the considerable overlap between GBRs and RBRs in the co-treatment, the relationships between binding of one factor and presence of the other were investigated. Of the 5,316 budesonide-induced GBRs not overlapping with an IL1B-induced RBR, 1,186 sites (22.3%) were lost with IL1B-plus-budesonide (Figure 2Di). Conversely, only 30 (2.6%) of the 1,163 budesonide-induced GBRs that overlapped with IL1B-induced RBRs were lost on co-treatment. Similarly, of the 3,575 IL1B-induced RBRs not showing budesonide-induced GBRs, 872 regions (24.4%) were lost with IL1B-plus-budesonide, while only 141 RBRs (4.5%) were lost from those overlapping GBRs (Figure 2Di). Thus, GR or RELA recruitment to the same region in response to budesonide or IL1B, respectively, correlates with protection from loss upon co-treatment.

The GR, or RELA, cistromes each gained 1,742, or 1,145, regions, respectively, with IL1B-plus-budesonide co-treatment (Figure 2A). With the GBRs induced by IL1B-plus-budesonide, only 447 (11.5%) of the 3,899 GBRs that did not recruit RELA were gained (Figure 2Dii). This compares with 1,295 (41.7%) of 3,106 GBRs that were gained where RELA was co-bound. Similarly, at the IL1B-plus-budesonide-induced RBRs, the 1,764 regions bound only by RELA gained only 95 (5.3%) RBRs, whereas RBRs that co-bound GR gained 1,050 (33.8%) new regions. Thus, GR, or RELA, presence at what become shared regions with IL1B-plus-budesonide favors gain of the other factor.

GBR and RBR loss and gain are readily discernible from binding intensity heatmaps of all binding regions centered on each peak alongside binding of the other factor to the same region (Figure 2E). Lost GBRs and RBRs showed little to no binding of the other factor, while gained regions generally revealed binding of the other factor in the respective mono-treatment that appeared to increase with co-treatment. At maintained GBRs and RBRs, the other factor was also clearly apparent (albeit often below the pre-specified threshold), and this increased with co-treatment. Thus, an overall positive correlation exists between GR and RELA binding with co-treatment (Figure S10). These data also reveal a general relationship between the presence, or intensity, of each factor and behavior of the other. GBRs that did not co-recruit RELA revealed significant GR reductions with IL1B-plus-budesonide, whereas GBRs that co-bound RELA significantly increased GR binding with IL1B-plus-budesonide (Figure 2F). Thus, differential GR-binding behavior comparing IL1B-plus-budesonide to budesonide correlated positively with IL1B-plus-budesonide-induced RELA intensity (Figure 2G). Hence, low or no RELA binding at a GBR predicts loss of GR on co-treatment, whereas high RELA binding predicts a gain in GR binding on co-treatment. Equivalent relationships were apparent for RBRs. IL1B-induced RBRs that did not recruit GR were reduced with IL1B-plus-budesonide, whereas RBRs that brought in GR were significantly increased by co-treatment (Figure 2F). Thus, differential RELA binding (IL1B-plus-budesonide/IL1B) at RBRs correlated positively with GR intensity on co-treatment (Figure 2G).

### GR and RELA binding each correlate with RNAP2

RNAP2 ChIP-seq was performed following 1 h of budesonide, IL1B, or IL-1B-plus-budesonide to assess their effects on RNAP2 recruitment. Budesonide increased RNAP2 at TSSs near various GBRs and/or along the gene bodies for known glucocorticoid-induced genes (*PER1*, *KLF9*, *MT2A*, *NFKBIA*, *TNFAIP3*, and *ZFP36*) (Figures S5A and S8A).^12^^,^^14^^,^^41^^,^^45^ Similarly, NF-κB-dependent genes (*CXCL8*, *NFKBIA*, *TNFAIP3*, and *ZFP36*) revealed RNAP2 peaks at the TSS, some RBRs, and often along the gene bodies in response to IL1B (Figures S5C and S8B).^30^^,^^46^ Furthermore, since IL1B-induced RELA and budesonide-induced GR recruitment each correlated positively with RNAP2, these co-recruitment patterns were also apparent genome-wide (Figure S11A). Similar correlations were also evident for IL1B-plus-budesonide (Figure S11B). Thus, while relationships between RNAP2 recruitment and gene transcription are complex,^47^^,^^48^ these data are consistent with GR and RELA enhancing RNAP2 recruitment in each of the three treatment groups.

### GBR behavior correlates with RELA intensity and effect on RNAP2 recruitment

**Figure 3:**
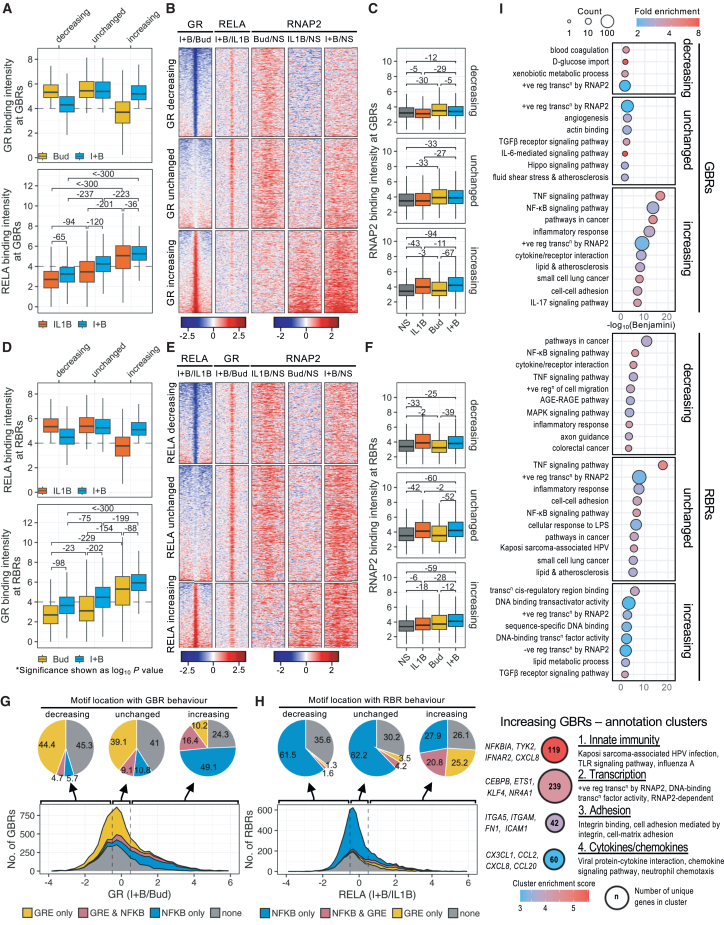
Binding site behavior correlates with RNAP2 enrichment and motif distribution A549 cells were treated as in Figure 1 for 1 h prior to RNAP2 ChIP-seq. *N* = 2 experiments. Data were combined with those from Figure 2. (A) Effect of treatment on GR (upper) or RELA (lower) binding intensity (log_2_-normalized read counts) for decreasing, unchanged, and increasing GBRs with co-treatment. (B) Heatmaps depicting log_2_ fold change in binding intensity of GR, RELA, and RNAP2 for the indicated treatments at GBRs, grouped by GR behavior. (C) Effect of treatment on RNAP2 binding intensity within GBR groups. (D) Effect of treatment on RELA (upper) or GR (lower) binding intensity for RBR groups. (E) Heatmaps depicting log_2_ fold change in binding intensity of RELA, GR, and RNAP2 for the indicated treatments at RBRs, grouped by RELA behavior. (F) Effect of treatment on RNAP2 binding intensity within RBR groups. (G) GRE and NF-κB motif distribution with GBR behavior. (H) NF-κB and GRE motif distribution with RBR behavior. (I) GO for genes ≤30 kb of GBRs and RBRs. (J) Four most enriched GO clusters for increasing GBRs. (A, C, D, and F) Data are presented as box-and-whiskers plots. (A and D) Data were analyzed using either Wilcoxon test for comparing between treatment within the same group or Mann-Whitney U test for comparing between groups; log_10_ derivatives of *p* values are depicted only when *p* ≤ 0.05. (C and F) Data were analyzed using Dunn’s test, and log_10_ derivatives of *p* values are indicated only when *p* ≤ 0.05.

Understanding that GBR and RBR behavior in the co-treatment represents a continuum of change-prompted redefinition of the GR and RELA cistrome heatmaps, with associated RNAP2, according to change on co-treatment (Figure S12). Regions where log_2_ differential GR binding (IL1B-plus-budesonide/budesonide) was ≤−0.5 were defined as “decreasing,” between >−0.5 and <0.5 were “unchanged,” and ≥0.5 “increasing” (Figure S12A). Using these GBR definitions as input variables, RELA binding intensity was tested as a dependent variable (Figure 3A). Decreasing GBRs were characterized by low RELA relative to unchanged or increasing regions. Unchanged GBRs revealed more RELA than decreasing regions, but less than increasing GBRs, while increasing GBRs revealed the most RELA. RELA intensity also increased within each group on co-treatment.

The GR cistrome, with associated RELA and RNAP2 binding (Figure S12A), was re-visualized to show differential GR and RELA binding between the combination and each mono-treatment along with RNAP2 fold change for each treatment (Figure 3B). At decreasing GBRs, reduced GR binding with IL1B-plus-budesonide was accompanied by modest RELA increases with co-treatment compared to IL1B, while budesonide-induced increases in RNAP2 were reduced on co-treatment (Figures 3B and 3C). This effect was consistent with reduced RNAP2 recruitment produced by IL1B and occurred despite IL1B-plus-budesonide modestly increasing low RELA levels (Figures 3A–3C; Figure S12A).

At unchanged GBRs, budesonide-induced GR recruitment associated with increased RNAP2, whereas IL1B was without effect (Figures 3B and 3C). However, although IL1B-plus-budesonide modestly increased RELA recruitment to these regions, there was no significant change in RNAP2 between budesonide and IL1B-plus-budesonide treatments (Figures 3A–3C).

At increasing GBRs, high RELA levels induced by IL1B and modest budesonide-induced GR binding both increased on combination treatment (Figures 3A, 3B, and S12A). Furthermore, each mono-treatment increased RNAP2 and IL1B-plus-budesonide further enhanced RNAP2 compared to either mono-treatment (Figures 3B and 3C). Thus, increasing GBRs are primarily IL1B-induced RBRs, where GR recruitment was enhanced and IL1B-induced RNAP2 recruitment was further increased by IL1B-plus-budesonide.

### RBR behavior correlates with GR intensity and effect on RNAP2 recruitment

The RELA cistrome heatmaps were also re-ordered according to the effect of IL1B-plus-budesonide compared to IL1B where log_2_ differential RELA binding (IL1B-plus-budesonide/IL1B) ≤−0.5 is “decreasing,” >−0.5/<0.5 is “unchanged,” and ≥0.5 is “increasing” (Figure S12B). Using RBR behavior as the input and GBR intensity as the dependent variable, decreasing RBRs showed lowest GR recruitment, unchanged RBRs showed intermediate GR, and gained RBRs revealed the most GR (Figure 3D). Thus, RELA behavior with IL1B-plus-budesonide predicts GR enrichment at that site. However, IL1B-plus-budesonide also significantly increased budesonide-induced GR recruitment for each group (Figure 3D).

At decreasing RBRs, IL1B-induced RELA associated with increased RNAP2 (Figures 3E and 3F). However, while IL1B-plus-budesonide reduced RELA recruitment, GR binding modestly increased with co-treatment relative to the low budesonide-induced GR binding (Figures 3D–3F). Thus, budesonide alone produced a small reduction in RNAP2 and, with IL1B-plus-budesonide, the modest RNAP2 loss compared to IL1B treatment did not reach significance (Figures 3E and 3F).

At unchanged RBRs, the modest budesonide-induced increase in GR was not associated with significant RNAP2 increases (Figures 3E and 3F). However, this effect was more prominent with co-treatment where IL1B-induced recruitment of RNAP2 was significantly enhanced by budesonide co-treatment (Figures 3E, 3F, and S12B).

Increasing RBRs showed low IL1B-induced RELA binding that was enhanced by budesonide combined with strong budesonide-induced GR binding that further increased with co-treatment (Figures 3D, 3E, and S12B). At these RBRs, IL1B- and budesonide-induced increases in RNAP2 were each significantly enhanced with IL1B-plus-budesonide (Figures 3E and 3F). Thus, increased RELA and GR recruitment following IL1B-plus-budesonide co-treatment was paralleled by increased RNAP2 recruitment.

### Binding region behavior correlates with differential motif presence

While MEME-ChIP identified GREs in 70% of GBRs and NF-κB motifs in 76% of RBRs, this necessarily captures many weak sites.^49^ Thus, focusing on motifs with JASPAR-defined scores ≥400 (i.e., *p* = 10^−4^), as representing strong motifs,^50^ showed 61.0% of RBRs with RELA, REL, or RELB motifs, and these were collectively defined as NF-κB motifs. More strikingly, only 41.6% of GBRs had JASPAR-defined consensus motifs for NR3C1 (GR) or mineralocorticoid (NR3C2) receptor, which were collectively defined as GREs. The distribution of these strong motifs was then explored in respect of RBR or GBR behavior (Figures 3G and 3H). Decreasing and unchanged GBRs preferentially showed strong GREs, with 49.1% and 48.2% of GBRs containing GRE motifs, of which 4.7% and 9.1% also revealed NF-κB motifs (Figure 3G). While this is consistent with GR binding at simple palindromic GREs, that more than half of GBRs do not show strong GREs suggests weaker motifs and/or alternative mechanisms of GR recruitment. Notably, only 26.6% of increasing GBRs contained GREs, with a majority (16.4%) being in conjunction with NF-κB motifs (Figure 3G). Further, with 49.1% of the increasing GBRs showing NF-κB motifs, but no GRE, most (65.5%) increasing GBRs contained NF-κB motifs (Figure 3G). While GREs and NF-κB motifs at increasing GBRs may enable conventional binding by each factor, the presence of GR at GBRs that contain NF-κB motifs, but no strong GRE motif, suggests other binding motifs and/or recruitment mechanisms for GR. Equivalent analysis of lost and gained GBRs confirmed the above trends (Figure S13). Thus, irrespective of GR-binding mode, increasing GBRs occurred at RBRs that strongly recruited RELA (Figure 3A), predominantly contained strong NF-κB motifs (Figure 3G), and showed increased RNAP2 relative to each mono-treatment and untreated (Figure 3C).

At decreasing and unchanged RBRs, 63.1% and 66.9% of regions contained strong NF-κB motifs (Figure 3H). Furthermore, with 1.6% and 4.2% containing both NF-κB and GRE motifs and similar low fractions showing only GRE motifs, these RBRs are consistent with RELA binding via classical NF-κB motifs. This contrasts with increasing RBRs, which showed a reduced fraction (48.7%) with NF-κB motifs (Figure 3H). Furthermore, 20.8% of increasing RBRs contained both NF-κB and GRE motifs and, most striking, 25.2% showed only strong GRE motifs. Thus, almost half the increasing RBRs showed strong GRE motifs such that strong NF-κB and strong GREs were similarly represented (Figure 3H). While presence of strong NF-κB motifs alone, or with GRE motifs, aligns with regulation by NF-κB and GR via conventional dimeric DNA binding, the majority of RBRs with only GREs or neither NF-κB motifs nor GREs suggests that RELA could be recruited by distinct mechanisms. Motif distribution in the lost and gained RBRs supports these findings (Figure S13). Thus, at increasing RBRs, RELA was generally recruited by budesonide co-addition, and while budesonide and IL1B each recruited RNAP2, co-treatment produced greater RNAP2 recruitment than either mono-treatment and these events variably involved strong NF-κB motifs and/or GREs (Figures 3D, 3F, and 3H).

### Inflammatory signature at RBRs and increasing GBRs

To explore the functional nature of the genes associated with GBRs or RBRs, Gene Ontology (GO) enrichment analysis was performed for those genes within 30 kb of GBRs or RBRs. For the RBRs, this revealed an overall inflammatory signature with decreasing RBRs associated with GO for NF-κB, cytokines, inflammation, and cancer, while unchanged RBRs strongly associated with inflammatory signaling and transcription (Figure 3I; Table S1A). The increasing RBRs revealed transcription as a main theme. GO for decreasing GBRs revealed metabolic terms, while unchanged GBRs associated with GO for transcription, TGFβ, IL-6, and hippo signaling. This contrasted with increasing GBRs, where a robust GO signature for inflammation involving NF-κB, TNFα, and IL-17 signaling, plus terms for cancer, was prominent (Figure 3I; Table S1A). These themes were reinforced by GO clustering, which emphasized innate immunity and viral and chemotactic responses for increasing GBRs (Figure 3J; Table S1A). These data suggest distinct functional roles in driving gene expression for the GBRs and RBRs that revealed differential binding behavior upon IL1B-plus-budesonide co-treatment.

### GR cistrome remodeling requires NF-κB

**Figure 4:**
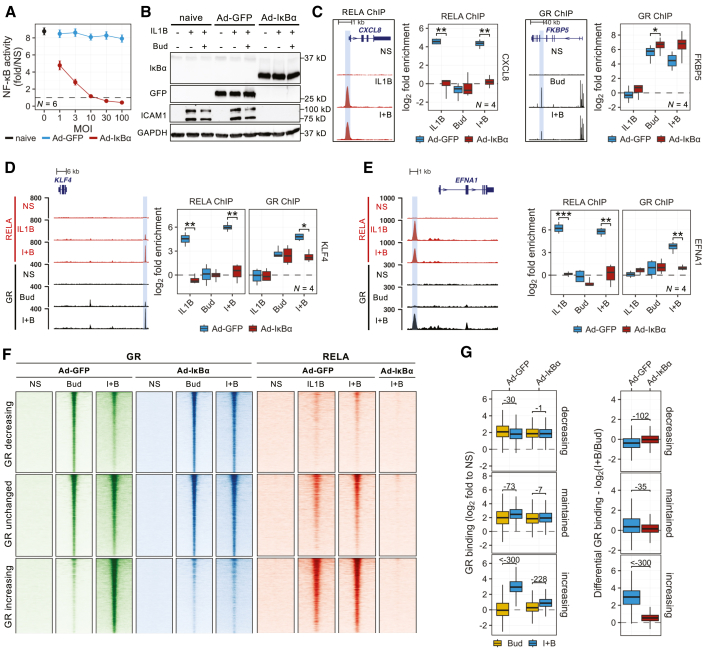
NF-κB inhibition prevents GR cistrome remodeling by IL1B-plus-budesonide (A) Effect of IL1B treatment on luciferase activity in NF-κB reporter cells infected with indicated MOIs of Ad5-IκBαΔN or Ad5-GFP. *N* = 6 experiments. Data are presented as means ± SE. (B) Effect of MOI 30 Ad5-IκBαΔN or Ad5-GFP on ICAM1 expression in A549 cells following IL1B or I + B for 2 h. *N* = 4 experiments. (C–G) A549 cells infected with MOI 30 Ad5-IκBαΔN, or Ad5-GFP, and harvested for GR and RELA ChIP 1 h after IL1B and/or Bud treatment. (C–E) Genome browser tracks (from Figure 2) highlighting target regions (left) for ChIP-qPCR (right, *N* = 4 experiments). Data are presented as box-and-whiskers plots and were analyzed using paired *t* test, where ∗, ∗∗, and ∗∗∗ represent *p* ≤ 0.05, *p* ≤ 0.01, and *p* ≤ 0.001, respectively. (F) Heatmaps depicting ChIP-seq binding intensity (log_2_-normalized read counts) for the indicated conditions/treatments for GR following GFP and IκBαΔN overexpression (*N* = 2 experiments, and RELA at all GBRs, grouped as in Figure 3). (G) Following Bud or I + B, GR binding (log_2_fold) (left) and fold-differential (log_2_I + B/Bud) (right) following GFP and IκBαΔN overexpression for each GBR group. Data presented as box-and-whiskers plots and was analyzed using Wilcoxon test; log_10_ derivatives of *p* values are indicated.

The role of NF-κB in mediating changes in the GR cistrome on co-treatment was assessed using adenoviral delivery of IκBαΔN, a dominant inhibitor of NF-κB DNA binding and transcription.^38^ This concentration-dependently prevented IL1B-induced NF-κB activation and mRNA expression of the NF-κB-dependent genes *CXCL8* and *ICAM1* (Figures 4A and S14A).^51^ At MOI of 30, GFP and IκBαΔN transduction was confirmed and IL1B- and IL1B-plus-budesonide-induced expression of ICAM1 and CXCL8 was prevented by IκBαΔN, but not GFP (Figures 4B and S14B). This confirms intervention effectiveness and paves the way to test effects on GR recruitment.

Cells transduced with Ad5-GFP, or Ad5-IκBαΔN, were treated with IL1B and/or budesonide for 1 h prior to RELA and GR ChIP. qPCR was used to confirm RELA recruitment to the 5′ *CXCL8* RBR in IL1B- and IL1B-plus-budesonide-treated cells transduced with GFP, but not IκBαΔN (Figure 4C). Conversely, the *FKBP5* locus recruited GR in Ad5-GFP- and Ad5-IκBαΔN-transduced cells treated with budesonide and IL1B-plus-budesonide (Figure 4C). These data confirm the loss of RELA recruitment at a well-characterized RBR and also show that general effects on GR recruitment are not likely. To explore the effect on GR recruitment, the increasing GBRs upstream of the *KLF4* and *EFNA1* genes were selected for further analysis.

Expression of *KLF4* shows cooperative IL1B-plus-budesonide-induced mRNA expression that depended on NF-κB (Figures S15A and S15B). Upstream of this gene is an increasing GBR with strong GRE and NF-κB motifs where IL1B-induced RELA recruitment and budesonide-induced GR recruitment were both enhanced by IL1B-plus-budesonide (Figure 4D). This was recapitulated in Ad5-GFP-infected cells, but with Ad5-IκBαΔN, there was no RELA enrichment and budesonide-induced GR was no longer enhanced by IL1B-plus-budesonide. The second increasing GBR, just 5′ to *EFNA1* was selected to represent IL1B-induced RBRs with robust NF-κB motifs, but no GREs (Figure S14C). *EFNA1* mRNA expression was repressed by budesonide alone and IL1B-induced mRNA, which was modestly reduced with IL1B-plus-budesonide, was strongly inhibited by IκBαΔN (Figures S15C and S15D). At this increasing GBR, GR was only recruited with the co-treatment (Figure 4E). ChIP-qPCR also confirmed that IL1B- and IL1B-plus-budesonide-induced RELA recruitment was abolished by Ad5-IκBαΔN, but not by Ad5-GFP. Furthermore, IL1B-plus-budesonide-induced GR recruitment was also absent with Ad5-IκBαΔN (Figure 4E). Thus, GR recruitment to both these “increasing” GBRs was dependent on NF-κB.

ChIP sequencing was next performed to more globally assess the effects of NF-κB inhibition on GR cistrome remodeling. Following Ad5-GFP infection, this analysis confirmed the decreasing and increasing GR enrichment patterns in the previously identified 8,221 GBRs, and these profiles again revealed low and high levels, respectively, of RELA recruitment (Figure 4F). With Ad5-IκBαΔN, there was a near-complete absence of RELA signal and the prior reduction in budesonide-induced GR observed at decreasing GBRs on co-treatment was prevented (Figures 4F and 4G). At increasing GBRs, where IL1B-induced RELA enrichment was high, the increased GR on IL1B-plus-budesonide co-treatment compared to budesonide was also prevented by IκBαΔN relative to GFP (Figures 4F and 4G). Thus, NF-κB was necessary for enhanced GR recruitment on co-treatment at these loci. The failure to recruit GR was readily apparent at the RBRs present in *C3*, *CXCL8*, *IL32*, *ICAM1*, and *SOD2* (Figure S16A). Likewise, at GBRs near *FKBP5*, *KLF9*, and *MT2A*, the repressive effects of IL1B on budesonide-induced GR recruitment were also abolished (Figure S16B).

### Budesonide and IL1B co-regulate gene expression

**Figure 5:**
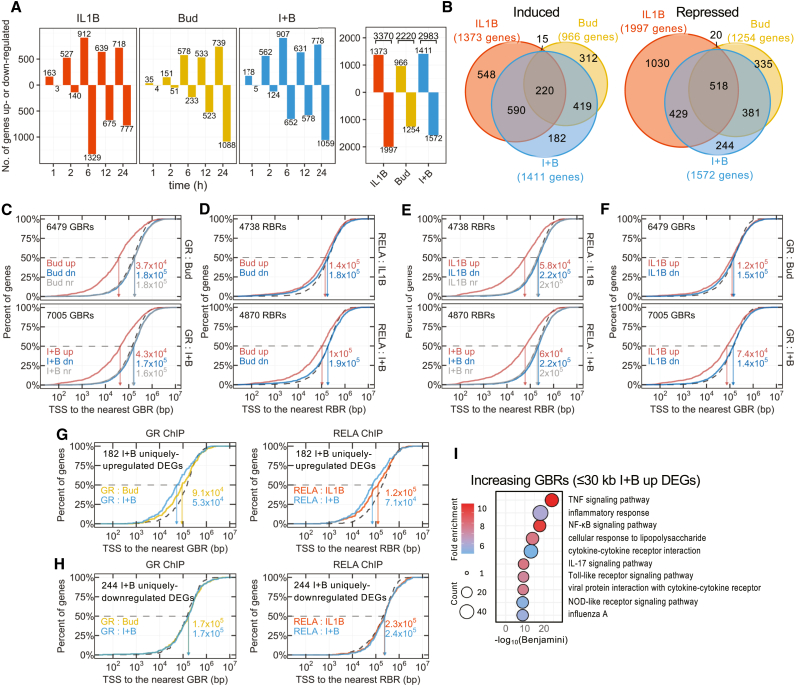
Binding regions associate with upregulated genes Cells treated as in Figure 1 were harvested at the indicated times for mRNA-seq. *N* = 4 experiments. (A) Number of up- or downregulated DEGs at each time (left) or in total (right) for each treatment. (B) Overlap between total DEGs with treatments. (C–F) Cumulative distribution of distances between TSS of DEGs up- (red), down- (dn; blue) or not regulated (nr; gray) by Bud, IL1B, or I + B and the nearest GBR or RBR in the indicated ChIP-seq dataset. TSS distance to equivalent numbers of random sites is shown (dotted line). (G and H) Distances between the TSS of genes uniquely up- (G) or downregulated (H) by I + B and the nearest GBR (left) or RBR (right) in the indicated ChIP-seq datasets. (I) GO for I + B-induced DEGs ≤30 kb of increasing GBRs.

To enable correlation of factor binding with possible changes in gene expression, A549 cells were treated with IL1B, budesonide, or both together for various times prior to mRNA sequencing. Analysis of differentially expressed genes (DEGs) (≥1/≤−1 log_2_ fold, false discovery rate ≤0.05) revealed increased DEG numbers induced by each treatment that preceded DEG repression (Figure 5A). Further, the number of DEGs regulated by IL1B (3,370) and budesonide (2,220) compared to IL1B-plus-budesonide (2,983) was strikingly less than additive and indicates co-regulation of gene expression (Figures 5A and 5B). Indeed, mutual up- and downregulation of DEGs by IL1B and budesonide was more frequent than chance (Figure S17A). Equally, many IL1B- and budesonide-regulated genes revealed opposing effects, which was more common than chance (Figure S17B). Thus, many IL1B- or budesonide-regulated DEGs failed to meet the criteria for regulation in the combination treatment, whereas other DEGs only met the criteria for regulation with IL1B-plus-budesonide (Figure 5B). Hence, relative to each mono-treatment, co-treatment produced DEG loss and gain such that the overall combined effects were not merely additive.

### GBR/RBR position correlates with upregulated DEGs

To explore the relationship between GBR or RBR and possible effects on gene expression, the distance from TSS to the nearest GBR or RBR was calculated in each ChIP-seq dataset for all expressed genes. Budesonide-induced GBRs mapped an average of 37 kb from budesonide-upregulated genes (Figure 5C, upper), a distance that was shorter (16 kb) for the more rapidly upregulated DEGs compared to those upregulated at the later times (41 kb at 24 h) (Figure S18A). This compares with the budesonide-downregulated and unregulated gene TSSs, which were on average ∼180 kb to the nearest GBR, a distance that was similar between TSSs of all expressed genes and equal numbers of random regions (Figure 5C, upper). Similarly, IL1B-plus-budesonide-induced DEGs revealed a closer average association (43 kb) with GBRs compared to unregulated and IL1B-plus-budesonide-repressed genes (∼160–170 kb) (Figure 5C, lower), values that were again similar for random regions. These data are, therefore, consistent with GBRs induced by budesonide, or IL1B-plus-budesonide, playing direct roles in upregulating associated DEGs. Conversely, no evidence was provided for direct GBR regulation of unregulated genes or budesonide-repressed DEGs.

Turning to possible associations between budesonide-induced and repressed DEG TSSs and the closest IL1B-induced RBR showed this to be similar to random regions (Figure 5D, upper). Thus, IL1B-activated RELA was not generally recruited to budesonide-regulated DEG loci. Nevertheless, the noticeably reduced TSS to IL1B-plus-budesonide-induced RBR distance for budesonide-upregulated DEGs suggests possible roles for RELA with the IL1B-plus-budesonide-upregulated DEGs (Figure 5D, lower).

Equivalent analyses for IL1B-regulated RBRs revealed an average TSS to nearest RBR distance of 58 kb for IL1B-upregulated DEGs that increased to ∼200 kb for IL1B-downregulated and unregulated genes, a distance that was not different from random regions (Figure 5E, upper). Further, the more rapidly IL1B-upregulated DEGs were more closed associated with RBRs (20 kb at 1 h) when compared to the IL1B-upregulated DEGs that were increased at later times (58–110 kb at 6–24 h) (Figure S18B). Similar data also occurred for IL1B-plus-budesonide-regulated DEGs with RBRs induced by IL1B-plus-budesonide (Figure 5E, lower). Thus, RELA recruitment correlates with increased IL1B- and IL1B-plus-budesonide-induced DEG expression. Conversely, no direct RELA role was suggested in the repression of DEGs by IL1B or IL1B-plus-budesonide.

While the distance between TSS of IL1B up- and downregulated DEGs and nearest GBR was similar to random regions (Figure 5F, upper), with IL1B-plus-budesonide-induced GBRs, a modest increase in GBR proximity to TSSs of IL1B-upregulated DEGs was apparent (Figure 5F, lower). Conversely, IL1B-repressed DEGs revealed little, or no, difference in the TSS-GBR distance compared to that for random regions. Therefore, with IL1B-plus-budesonide, roles for GR in up-regulating IL1B-induced genes are supported.

### GBR/RBR association with DEGs only upregulated by IL1B-plus-budesonide

The 182 and 244 DEGs up- or downregulated, respectively, (Figure 5B), only by IL1B-plus-budesonide, revealed weak TSS to GBR or RBR associations between co-upregulated genes in each respective mono-treatment that were enhanced by co-treatment (Figure 5G). Whereas these data are consistent with both factors playing direct roles in co-upregulation, no association was apparent with co-downregulated DEG loci (Figure 5H).

### RBRs and increasing GBRs near upregulated DEGs reveal inflammatory GO

To gain improved insight as to the possible downstream functions of the DEGs mostly likely to be regulated by GR and RELA, the gene lists showing ≤30 kb proximity to GBRs or RBRs were filtered by those DEGs upregulated in the relevant treatment. This confirmed a robust inflammatory GO signal for all three RBR groups (Tables S2A and S2B). While decreasing and unchanged GBRs produced weak signatures, the increasing GBRs near IL1B-plus-budesonide-induced DEGs yielded a striking inflammatory GO signature that included terms for inflammation, viral infection, and TNFα, IL-17, NOD, and TLR signaling (Figure 5I; Tables S2A and S2B).

### RBRs may protect budesonide-upregulated DEGs from expression loss on co-treatment

**Figure 6:**
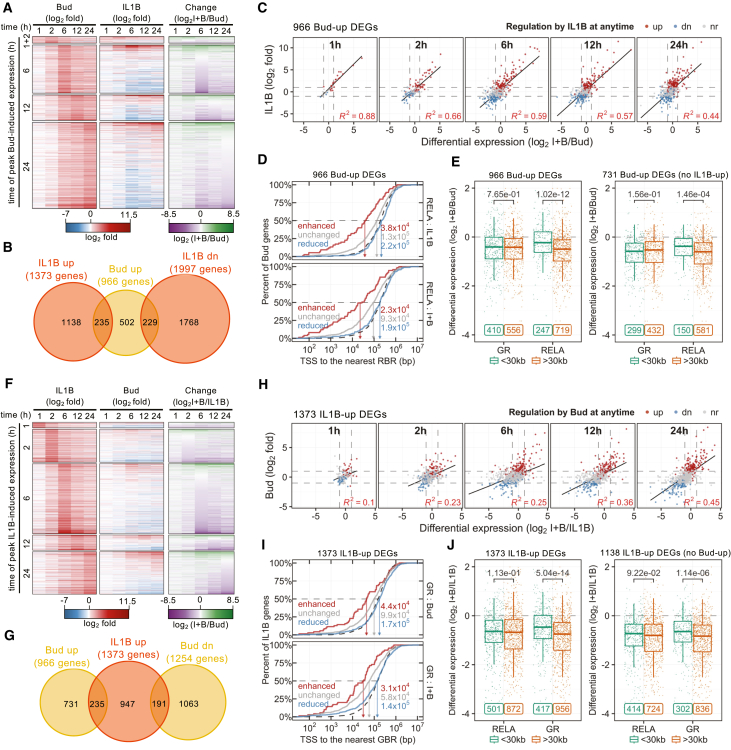
Relationship between upregulated DEG behavior with IL1B-plus-budesonide and GBR and RBR proximity (A) Heatmaps for all Bud-upregulated DEGs grouped by time of peak induction showing effect of Bud and IL1B (log_2_fold) ranked by change on co-treatment (log_2_(I + B/Bud)). (B) Overlap between the Bud-upregulated DEGs and DEGs up- or downregulated by IL1B. (C) Effect of IL1B plotted against change on co-treatment for budesonide-induced DEGs, grouped by time of peak induction. (D) Cumulative distribution of the distances between TSS of DEGs enhanced (red), reduced (blue), or unchanged (gray) by I + B, compared to Bud, and the nearest RBR in the indicated RELA ChIP-seq dataset. TSS distance to equivalent numbers of random sites is shown (dotted line). (E) Relationship between proximity (≤30 or >30 kb) to GBRs or RBRs on expression change with co-treatment for all Bud-upregulated DEGs (left) or Bud-upregulated DEGs not independently upregulated by IL1B (right). Individual observations are also plotted as scattered points. Data presented as box-and-whiskers plots and was analyzed using Mann-Whitney U test; numeric *p* values are depicted. (F–J) Similar to (A–E), figures show effect of Bud on IL1B-induced genes and relationships with GR binding.

Given the increased presence of RELA at many GBRs, studies were carried out to assess possible correlations with gene expression. Grouping the 966 budesonide-upregulated DEGs by peak response time and then ranking by the effect of IL1B-plus-budesonide/budesonide revealed budesonide inducibility that was often combined with up- or down-modulation by IL1B as a co-treatment (Figure 6A). Furthermore, IL1B independently up- and downregulated 464 of the budesonide-upregulated DEGs (Figures 6A and 6B), an effect that was highly predictive (r^2^ = 0.88–0.44) of budesonide-induced DEG behavior with IL1B-plus-budesonide (Figure 6C). Thus, budesonide-upregulated DEGs that were independently IL1B-upregulated were more likely to be further enhanced with IL1B-plus-budesonide. Conversely, budesonide-upregulated DEGs showing independent downregulation by IL1B were more likely to be reduced, relative to budesonide, by co-treatment.

The effect of IL1B on budesonide-induced DEGs was, therefore, examined relative to RBR location as a possible means of explaining DEG behavior in the combination treatment. This analysis revealed that the TSSs of budesonide-upregulated DEGs showing enhanced expression with co-treatment (i.e., log_2_ I + B/Bud≥1) were closer to IL1B-induced RBRs (average 38 kb) than those showing loss of expression (log_2_ I + B/Bud ≤ −1) with co-treatment (average 220 kb) (Figure 6D, upper). This was more pronounced with IL1B-plus-budesonide-induced RBRs, which revealed an average TSS-RBR distance of 23 kb for the budesonide-upregulated genes that were further enhanced by IL1B co-treatment (Figure 6D, lower). Conversely, TSS-RBR distances for the budesonide-upregulated genes that show expression loses with IL1B co-treatment were similar to that for random regions. Thus, proximity of budesonide-induced DEG TSSs to RBRs was associated with increased expression with co-treatment, but there was no evidence to support direct *cis*-acting effects of RELA in repressing budesonide-upregulated DEGs. Furthermore, TSSs of budesonide-upregulated genes that were unchanged (log_2_ I + B/Bud<1/>−1) by IL1B co-treatment were closer to RBRs than either that for DEGs repressed by IL1B co-treatment or the TSS to random regions. This suggests that budesonide-induced DEGs that were unchanged by IL1B co-treatment may also be directly regulated by RELA, a concept consistent with many of these DEGs showing independent, albeit often modest, upregulation by IL1B (Figures 6A–6C).

To further explore relationships between GBR and RBR location and losses of budesonide-upregulated gene expression with IL1B-plus-budesonide, budesonide-induced DEG behavior was categorized by TSS closeness (≤30 kb) to GBRs and RBRs. This revealed that the overall net repressive effect of IL1B on budesonide-upregulated DEGs was independent of GBR proximity (Figure 6E, left). However, while budesonide-upregulated DEGs that were >30 kb from an RBR showed reductions in expression with IL1B co-treatment relative to budesonide alone, those DEGs closer to (≤30 kb) an RBR were significantly less reduced by the co-treatment (Figure 6E, left). This association also held true in respect of the DEGs that were upregulated by budesonide at each individual time point (Figure S19A). Further, the data reflective of a spectrum of effect, whereby distance from TSS of budesonide-upregulated DEGs to the nearest GBR showed no impact on repression but distance to nearest RBR inversely correlated with loss due to IL1B in the context of budesonide (Figure S19B). Collectively, these data support roles for RELA acting directly at each DEG locus to enhance/maintain expression of budesonide-upregulated DEGs upon IL1B co-treatment. Thus, protection against the net reduction in budesonide-induced expression with IL1B-plus-budesonide was most apparent where GBRs were close to the regulated DEG TSS and the effect occurred even where budesonide inducibility was markedly delayed. Since many budesonide-upregulated DEGs were independently upregulated by IL1B (Figures 6A–6C), such a relationship was perhaps unsurprising. However, after excluding the 235 budesonide-upregulated DEGs showing independent upregulation by IL1B, RBR presence ≤30 kb of TSSs still associated with protection against repression in the combination treatment (Figure 6E, right). Finally, to assess the role of RELA when recruited to those budesonide-upregulated DEGs that were most likely to be directly controlled by GR, the 410 budesonide-upregulated DEGs from Figure 6E were filtered according to RELA proximity (Figure S19C). This analysis confirmed that the net loss of expression for the 410 budesonide-upregulated DEGs in the co-treatment was significantly attenuated when close to (≤30 kb) an RBR. This, therefore, supports the concept that RELA presence may protect from a loss of budesonide-induced upregulation in the context of IL1B-plus-budesonide co-treatment.

### GBRs may protect IL1B-upregulated DEGs from expression loss on co-treatment

To explore possible roles for GR when recruited to RBRs, the 1,373 IL1B-upregulated DEGs were grouped by peak expression time and ranked by IL1B-plus-budesonide/IL1B. This revealed marked up- and down-modulation of the IL1B response by budesonide as a co-treatment (Figure 6F). Equally, independent regulation of 426 IL1B-induced DEGs by budesonide alone was clear, and as shown by positive correlations, this was predictive of outcomes in the combination treatment (Figures 6F–6H). Consistent with this, the IL1B-upregulated DEGs that were enhanced by budesonide co-treatment also showed lesser TSS-GBR distances that were, therefore, consistent with some degree of direct regulation by GR in both the budesonide and IL1B-plus-budesonide treatments (Figure 6I). Conversely, this effect was not apparent for IL1B-upregulated DEGs that were repressed by budesonide co-treatment. Rather, these TSS-GBR distances were similar to the TSS to random regions for both IL1B- and IL1B-plus-budesonide-induced GBRs, and thus no evidence for direct regulation by GR is provided (Figure 6I). Equally, IL1B-upregulated DEGs that were unaffected by budesonide in the combination treatment were also closer to GBRs compared to the random regions in both budesonide and IL1B-plus-budesonide ChIP analyses (Figure 6I). Thus, GBR presence near IL1B-upregulated DEGs may contribute to their further enhancement or, where IL1B-induced expression was unaffected by budesonide co-treatment, help maintain DEG expression. Importantly, these data provide no general evidence of direct roles for GR in mediating losses of IL1B-upregulated DEG expression in the IL1B-plus-budesonide co-treatment.

Formal testing confirmed that while RBR closeness (≤30 kb) was unrelated to loss of IL1B-upregulated DEG expression produced by budesonide in the co-treatment, having a close GBR, i.e., ≤30 kb from TSS, correlated with protection from expression loss on IL1B-plus-budesonide co-treatment (Figure 6J, left). Furthermore, this protective association with GR was replicated in respect of the IL1B-upregulated DEGs that were increased at each time point (1, 2, 6, 12, and 24 h) (Figure S19D). In addition, the association revealed a general inverse correlation with GBR distance from the TSS of the IL1B-upregulated gene (Figure S19E). This finding would reasonably be expected for any factor exerting a positive effect on gene transcription. Since this effect could be driven by those IL1B-upregulated DEGs that were independently upregulated by budesonide, the analysis was repeated following exclusion of the 235 budesonide-upregulated DEGs. This confirmed that reduced expression of IL1B-upregulated DEGs in the combination treatment was again independent of RBR proximity, but GBR proximity (≤30 kb) remained protective from loss in the co-treatment (Figure 6J, right). These data strongly support the notion that, rather than mediating direct repression of IL1B-upregulated DEGs, GR acts, possibly at, or near to, the gene locus to enhance or maintain expression of IL1B-upregulated DEGs in the context of IL1B-plus-budesonide. To more specifically extend this observation to those DEGs that were more likely to show upregulation by RELA, the 501 DEGs that were ≤30 kb from RBSs in Figure 6J were further filtered by proximity to the nearest GBR (Figure S19F). This analysis again confirmed that TSS proximity (≤30 kb) to a GBR associated with significantly attenuated expression loss on co-treatment compared to those DEGs that were further from GBRs. Taken together, these data suggest that, rather than broadly mediating reductions in IL1B-upregulated DEGs controlled by RELA/NF-κB during IL1B-plus-budesonide co-treatment, GR proximity may actually play a role in supporting the maintenance of IL1B-upregulated gene expression.

## Discussion

NF-κB regulates hundreds of inflammatory genes and is widely perceived as a key target for GR-mediated repression.^1^^,^^52^ In the current study, IL1B promoted RELA/NF-κB co-recruitment to numerous loci, many of which showed glucocorticoid-dependent GR recruitment that was enabled, or enhanced, by IL1B-plus-glucocorticoid. Described as increasing GBRs, these regions typically revealed robust RELA recruitment and strong NF-κB motifs, while a relative paucity of GRE motifs means “tethering” could explain GR recruitment.^13^^,^^25^^,^^53^ This would link with concepts of tethered transrepression, whereby GR interaction with NF-κB may elicit transcriptional repression.^1^^,^^2^ Alternatively, while GR binding to DNA motifs may represent a separate mechanism of direct transrepression,^23^^,^^24^^,^^54^ it is pertinent that in both cases the genomic presence of GR should correlate with glucocorticoid-dependent repression. This, despite glucocorticoids causing GR-dependent loss of IL1B-upregulated gene expression, was not apparent in the current analysis.^30^

In considering NF-κB as a target for glucocorticoid-mediated repression, it is salient that although many IL1B-induced mRNAs were glucocorticoid-repressed, others were not and yet both groups were largely NF-κB-dependent in this model.^30^ However, despite this unclear relationship between NF-κB and glucocorticoid-dependent repression, nanometer-scale GR/RELA co-localization occurred in the nuclei of budesonide-plus-IL1B-treated cells. Indeed, while summit-summit distances for many overlapping GBR/RBE were consistent with possible direct interaction, others are less readily accounted for by direct GR-RELA interaction. Nevertheless, most RBRs mapped close to GBRs on IL1B-plus-budesonide treatment and similar NF-κB/GR associations occur in ChIP data from multiple studies.^13^^,^^14^^,^^15^^,^^16^^,^^53^ Thus, co-recruitment to common genomic regions is prevalent, but raises questions of function. In exploring this, GR or RELA recruitment following each mono-treatment and the co-treatment not only correlated with RNAP2 presence but also with upregulated DEGs. Thus, the genomic presence of each factor appears to positively regulate gene transcription. Further, the glucocorticoid-induced GR cistrome and IL1B-induced RELA cistrome each revealed losses and gains on co-treatment. Here, the overlapping presence of each factor in the mono-treatments correlated with protection from loss on co-treatment and co-recruitment with co-treatment favored gains in each factor. Thus, reciprocal, and mutual, cistrome remodeling produced considerable GBR/RBR overlap that correlated with RNAP2 presence. Further, GBRs or RBRs that were reduced on co-treatment primarily showed GRE or NF-κB motifs, respectively, were associated with low levels of the other factor, and did not show enhanced RNAP2 recruitment. Conversely, the increasing GBRs and RBRs showed an over-representation of motifs for, and high levels of, the other factor. As these regions showed enhanced RNAP2 recruitment in the co-treatment, correspondingly increased transcription of associated genes was expected, but may clearly be subject to additional control mechanisms.^55^

The concept that GR and RELA recruitment each associates with increased RNAP2 presence and upregulated DEG loci in the relevant mono-treatment or IL1B-plus-budesonide co-treatment fits well with existing models of transcriptional activation.^1^^,^^52^ Equally, the fact that RBRs, or GBRs, associated no more frequently with downregulated gene loci than random regions in respect of each mono-treatment or the co-treatment is consistent with other studies.^12^^,^^14^^,^^16^ Such data also argue against generalized mechanisms of direct repression by either factor, and this implicates indirect mechanisms for repression.^3^^,^^17^^,^^19^ However, RELA or GR presence at loci where the associated DEG shows expression loss in the co-treatment relative to the relevant mono-treatment is less intuitive. In exploring this phenomenon, independent up- or downregulation by IL1B was found to predict budesonide-upregulated DEG behavior in the combination treatment. Thus, budesonide-upregulated DEGs showing enhancement by IL1B were closer to RBRs than the unregulated DEGs or DEGs that lost expression. This supports positive regulation by RELA. Conversely, loss of budesonide-induced DEG expression on co-treatment did not associate with RBRs any more than for random regions. Hence no support for direct repression by RELA is provided. Indeed, loss of budesonide-induced DEG expression with IL1B as co-treatment was less pronounced where RBRs were close (≤30 kb) to the TSS compared to where RBRs were further away. As this effect extended to those budesonide-induced DEGs that were not independently regulated by IL1B, RELA co-recruitment appears protective from IL1B-mediated repression. Furthermore, the protective effect of RBR-TSS proximity held true in respect of budesonide-upregulated DEGs at each time from 1 to 24 h. This is consistent with glucocorticoids rapidly inducing both GR recruitment and long-range chromatin interactions that were stable, albeit likely dynamic,^56^ over extended time frames to regulate rapidly induced, as well as the more delayed phases of glucocorticoid-induced gene expression.^39^^,^^57^^,^^58^ Further, while RELA presence may also be stable with time, further studies are needed to investigate this.

Similar to above, 31% of the IL1B-upregulated DEGs were independently regulated by budesonide, and this effect was predictive of combination treatment-induced expression change. Thus, loci for IL1B-induced DEGs showing enhancement by, or even no effect of, budesonide in the co-treatment associated more closely with GBRs than the IL1B-upregulated DEGs that were reduced on co-treatment. Indeed, as IL1B-upregulated DEGs that were reduced by combination treatment were no closer to GBRs than to random regions, these data provide no general support for direct GR-mediated repression at these loci. Accordingly, whereas IL1B-induced DEG proximity to RBRs did not impact glucocorticoid-dependent repression on co-treatment, GBR proximity (≤30 kb) identified DEGs that were overall less repressed relative to those DEGs where the TSS was more distant from GBRs. That this persisted following exclusion of DEGs with independent glucocorticoid regulation reveals GR recruitment to IL1B-upregulated DEG loci as protecting against a wider, more prevalent, and presumably independent, GC-mediated repression. Further, in spite of ChIP analysis being performed 1 h after treatment, the protective effect of GBRs was apparent in respect of IL1B-upregulated DEGs at each individual time (1–24 h). This suggests that rather than transcriptional effects only being mediated at 1 h, RELA recruitment to DNA may, along with close GBRs, persist over prolonged periods of time as is reported for direct GR regulation.^39^^,^^57^^,^^58^ Equally, the data do not support GC-mediated repression of IL-1β-upregulated DEGs as being directly due to GR recruitment. Rather, indirect mechanisms by which GR mediates loss of IL1B-upregulated gene expression are suggested.

While IL1B regulation predicted effects of co-treatment on budesonide-upregulated DEGs, independent regulation by budesonide was less predictive of outcomes for IL1B-upregulated DEGs. Reasons may be various, but combination effects on IL1B-upregulated DEGs cannot be simply explained by glucocorticoid alone; there is permissiveness. For example, IL1B-upregulated pathways and DEGs that were not active, or expressed, until induced by IL1B, would only show glucocorticoid-dependent inhibition in the co-treatment. Equally, synergies or events only occurring with IL1B-plus-glucocorticoid may impact specific IL1B-upregulated DEGs. Such observations are pertinent since glucocorticoid-mediated repression of IL1B-upregulated DEGs is time-dependent and can be prevented by blocking gene expression.^30^ Thus, glucocorticoid-IL1B synergy, at DEGs that are increasingly recognized as promoting repression of IL1B-mediated gene expression, could explain the current data.^11^^,^^14^^,^^15^^,^^17^

Although GR/RELA cistrome remodeling was widespread, IL1B-induced GR cistrome remodeling was prevented by IκBαΔN to reveal a causal role for NF-κB. However, as decreasing GBRs revealed no, or low, RELA presence, direct NF-κB action is hard to reconcile. Putting aside possible rapid effects involving gene expression changes of modulatory factors, reduced GR recruitment may be accounted for by mass action consequent on GR gains at increasing GBRs, an effect that then requires explanation. Where increasing GBRs and RBRs overlap and contain both NF-κB and GRE motifs (e.g., *KLF4*), NF-κB, or GR, presence could be permissive. For example, by acting as a pioneer factor, RELA or GR could each promote chromatin remodeling to facilitate recruitment of the other.^59^^,^^60^^,^^61^^,^^62^^,^^63^ However, most increasing GBRs have no strong GREs, but rather show only NF-κB motifs where RELA presence raises the possibility that GR tethers via NF-κB to the DNA. Equally, tethering via other factors also remains possible. However, unconventional GR binding via half-, or hemi-, motifs will not appear in standard motif analyses, and thus, GR binding as a monomer,^64^ potentially along with other factors that could stabilize GR binding to DNA, remains possible.^65^ Since recruitment without apparent consensus motifs is common in ChIP-seq data and may be produced by DNA looping,^57^^,^^58^^,^^66^ or occurs at distances that are poorly consistent with direct interaction, simple assumptions of GR-RELA tethering are premature and require further investigation.

Adopting the proposition that GR, when recruited to IL1B-upregulated DEGs, protects against independent glucocorticoid-mediated repression requires consideration of biological rationale. One obvious aspect is the previously mentioned GR-RELA/NF-κB cooperation to enhance expression of genes, including *IRAK3*, that play repressive roles that could then affect numerous IL1B-upregulated DEGs.^11^^,^^14^^,^^15^^,^^17^ Equally, positive NF-κB/GR synergy at inflammatory genes, for example, *TLR2*^67^ or serum amyloid A,^68^ could play essential roles in innate immunity and host defense.^69^ Similarly, enhanced GR/RELA recruitment at genomic sites, without change in overall nuclear localization, should, as mentioned above, concomitantly reduce recruitment and associated transcription at other loci. Thus, a necessary counter-balancing, or tuning, mechanism could be the co-recruitment of both factors to ensure maintained expression on co-treatment. Indeed, such mechanisms are demonstrated in simple reporter systems.^70^ Equally, regulator loci including *NFKBIA* (IκBα) and *TNFAIP3* show RBRs and GBRs, with their DEGs being IL1B-induced and modestly glucocorticoid-induced mRNAs (Figure S5). These transcripts yielded no change from IL1B-induced expression on combination treatment, and thus downstream regulatory control should be maintained. Likewise, increasing GBRs at the IL1B-induced RBRs in *IL32*, an IL1B-induced DEG, could plausibly help maintain cytokine expression (Figure S8). Similarly, as shown by GO, GR co-recruitment with NF-κB occurs at anti-apoptotic and protective genes, including *BIRC3*,^43^ *CFLAR* (also known as c-FLIP), and *SOD2*, where their maintained expression is likely to be critical for cell survival (Figure S8). However, inflammatory genes, including *CXCL8* and *ICAM1*, show various degrees of glucocorticoid-dependent repression, yet also recruit GR to RBRs. In such instances, roles for GR recruitment are more contentious. Indeed, while prior paradigms would suggest that such GR recruitment was responsible for mediating repression,^1^ there was no general support for this mechanism in the current data. However, any association between factor binding to DNA and gene expression change is probabilistic and while being a genome-wide reality, may not apply at any given locus. Nevertheless, possible losses in coactivator presence, gains in co-repressors, changes in chromatin or RNAP2 serine 2 phosphorylation, or indeed other mechanisms to reduce transcriptional activation may all relate to the repressive effects of glucocorticoids and will be mediated via GR.^25^^,^^71^^,^^72^^,^^73^^,^^74^^,^^75^ However, these repressive events need not be direct effects of GR and most studies do not overtly distinguish direct from indirect effects of GR. Thus, in the context of events leading to general loss of IL1B-upregulated gene expression, the positioning of GR at any given locus could plausibly offset the more general repressive effect. This effect could, for example, play roles in delaying the onset of the indirect glucocorticoid-induced repression at specific genes. This novel concept will need to be explored in future studies, particularly to test the generalizability of the current data in primary cells relevant to inflammatory disease. However, GR/RELA interactions can positively regulate gene expression in primary airway epithelial cells via GBR and RBR that were also common to A549 cells.^11^^,^^67^ Given glucocorticoid- and inflammatory stimulus-regulated gene expression may be common to A549 cells and primary airway epithelial cells,^31^^,^^76^^,^^77^^,^^78^ the current data are likely to be relevant in both systems. Furthermore, the widespread reporting of GR/RELA interactions,^13^^,^^14^^,^^16^^,^^79^ including inflammatory cells,^15^ suggests wide relevance.

Based on the current analyses, we suggest that “local” GR recruitment to inflammatory genes is more generally consistent with enhanced, or maintained, expression, rather than directly causing repression. Furthermore, this new appreciation of the possible GR role should enable more appropriate discussions in respect of biological function, including in disease. Indeed, GO for genes associated with increasing GBRs revealed inflammatory signatures that align with molecular endotypes in glucocorticoid-resistant disease.^80^^,^^81^ We, therefore, submit that while GR recruitment to pro-inflammatory gene loci may, by helping to maintain gene expression, be important for host defense or repair,^69^ this effect could also be responsible for reduced responses to glucocorticoid where such genes are also important for driving pathology.

### Limitations of the study

Key limitations to the current analysis include the inability to determine whether GR and RELA are physically in contact or are merely in close proximity to each other. Here, both the imaging and the ChIP-seq data can only document close proximity between GR and RELA. As noted above, presence of RNAP2 is an imprecise index of associated transcription due to both the existence of additional regulatory control mechanisms and the failure to capture the dynamic nature of RNAP2 function. Thus, increased RNAP2 presence due to stalling is indistinguishable from increases in transcriptionally active RNAP2. An important caveat to the above findings is that the observations are based on the collective analysis of numerous binding regions and/or genes that may not necessarily reflect changes or regulation of individual DNA regions or genes. Thus, analysis of specific regions and their ability to regulate particular genes is critically important. Further, the relationship between GR and/or RELA at one time point on later gene expression needs in-depth examination. Equally, the reductionist approach of examining only GR and NF-κB/RELA will necessarily need to evolve into more holistic analyses that takes into account multiple factors, chromatin modification, and three-dimensional/spatial considerations that may collectively control gene expression. Finally, the current analysis is restricted to a single cell line. While features such as the ability of glucocorticoids acting via GR, or IL1β acting via NF-κB to up- and downregulate gene expression are widely observed, exploring many of the more detailed findings in additional models, including in primary human cells, will be important to evaluate the general applicability of the current findings.

## Resource availability

### Lead contact

Requests for additional information or resources and reagents should be directed to the lead contact, Robert Newton (rnewton@ucalgary.ca).

### Materials availability

This study did not generate new unique reagents.

### Data and code availability

• ChIP-seq and RNA-seq data have been deposited at GEO and are publicly available as of the date of publication. Accession numbers are listed in the key resources table.
• This paper does not report original code.
• Any additional information required to reanalyze the data reported in this paper is available from the lead contact upon request.

## Acknowledgments

This work was supported by grants from the 10.13039/501100000024Canadian Institutes of Health Research (PJT 156310 and 180480, both to R.N.) and 10.13039/501100000038Natural Sciences and Engineering Research Council of Canada discovery grants (RGPIN-2016-04549 and RGPIN-2023-03763, both to R.N.) and by studentships from Alberta Graduate Excellence Scholarships (ANB), Eyes High Doctoral Scholarship and Eleanor Mackie Doctoral Scholarship in Women’s Health from University of Calgary (AB), and 10.13039/100012866Cumming School of Medicine Graduate Scholarship (AG). Imaging was carried out at the Live Cell Imaging Laboratory, University of Calgary (RRID:SCR_024748).

## Author contributions

M.M.M. conceptualized, designed, performed, and analyzed experiments; prepared figures; and co-wrote the paper. A.N.-B., A.J.T., A.G., A.B., and A.M.M. performed and/or analyzed experiments, prepared figures, and reviewed the manuscript. P.C. and L.S. acquired microscopy images, supported image analysis, and reviewed the manuscript. S.K.S. and A.N.G. provided reagents and resources, analyzed experiments, and reviewed/edited the manuscript. R.N. acquired funding, conceptualized the project, designed and analyzed experiments, prepared figures, and drafted the manuscript.

## Declaration of interests

The authors declare no competing interests.

## STAR★Methods

### Key resources table

**Table:** 

REAGENT or RESOURCE	SOURCE	IDENTIFIER
**Antibodies**		

Mouse monoclonal anti-GR	Cell Signaling	Cat#: 47411S; RRID: AB_2799324
Rabbit monoclonal anti-RELA	Cell Signaling	Cat#: 8242S; RRID: AB_10859369
Mouse monoclonal anti- IκBα	Cell signaling	Cat#: 4814S; RRID: AB_390781
Rabbit monoclonal anti-EP300	Cell Signaling	Cat#: 86377S; RRID: AB_2800077
Rabbit polyclonal anti-GR	Sasse et al.^16^	GR-356
Rabbit polyclonal anti-GFP	Cell Signaling	Cat: 2555S; RRID: AB_10692764
Mouse monoclonal anti-GAPDH	BioRad	Cat#: MCA4739; RRID: AB_1720065
Rabbit monoclonal anti-CREB	Cell Signaling	Cat#: 9197S; RRID: AB_331277
Mouse monoclonal anti-ICAM1	Santa Cruz	Cat#: sc-8439; RRID: AB_627123
Anti-RPB1 NTD (RNA polymerase II subunit A)	Cell Signaling	Cat#: 14958S; RRID: AB_2687876
Goat anti-rabbit, HRP linked	Jackson ImmunoResearch	Cat#: 111-035-003; RRID: AB_2313567
Goat anti-mouse, HRP linked	Jackson ImmunoResearch	Cat#: 115-035-003; RRID: AB_10015289
Goat anti-rabbit, AF594 linked	Invitrogen	Cat#: A-11037; RRID: AB_2534095
Goat anti-mouse, AF594 linked	Invitrogen	Cat#: A-11032; RRID: AB_2534091
Goat anti-mouse, STAR-RED linked	abberior	Cat#: STRED-1001; RRID: AB_3068620
Donkey anti-Mouse, MINUS PLA Probe linked	Sigma-Aldrich	Cat#: DUO92004; RRID: AB_2713942
Donkey anti-Rabbit, PLUS PLA Probe linked	Sigma-Aldrich	Cat#: DUO92002; RRID: AB_2810940

**Bacterial and virus strains**		

Ad5-IκBαΔN	Catley et al.^38^	NA

**Chemicals, peptides, and recombinant proteins**		

DMEM	Invitrogen	11995073
Fatal Bovine serum	Invitrogen	A3160702
HBSS	Invitrogen	14175079
PBS	Invitrogen	14190250
L-Glutamine	Invitrogen	25030081
G418	Sigma-Aldrich	A1720
Budesonide	AstraZeneca	gift
Recombinant human IL-1b	R&D systems	201-LB-005
Actinomycin D	Sigma-Aldrich	A410
Quant-iT PicoGreen dsDNA Reagent	Invitrogen	P7581
16% Paraformaldehyde (w/v), Methanol-free	Thermo Scientific	043368
Dynabeads Protein G	Thermo	10004D
Proteinase K	Cell Signaling	10012S
RNase A	Cell Signaling	7013S
BlockAid Blocking Solution	Invitrogen	B10710
Image-iT FX Signal Enhancer	Invitrogen	I36933
ProLong Gold Antifade Mountant with DAPI	Invitrogen	P36941
ProLong Gold Antifade Mountant	Invitrogen	P10144
Halt Protease Inhibitor Cocktail 100x	Thermo Scientific	PI-78439
PMSF	Cell Signaling	8553S
Fast SYBR Green Master Mix	Thermo Scientific	4385618

**Critical commercial assays**		

Firefly Luciferase Assay Kit 2.0	Biotium	30085
NE-PER Nuclear and Cytoplasmic Extraction kit	Thermo Scientific	78833
ChIP DNA Clean & Concentrator	Zymo Research	D5205
NucleoSpin RNA purification kit	Macherey-Nagel	740955
Duolink *In Situ* Detection Reagents Red	Sigma-Aldrich	DUO92008
Duolink *In Situ* Wash Buffers, Fluorescence	Sigma-Aldrich	DUO82049
qScript cDNA Synthesis Kit	QuantaBio	95047

**Deposited data**		

A549 RNAseq	This paper	GEO: GSE295743
A549 GR and RELA ChIPseq	This paper	GEO: GSE296100
A549 RNAP2 ChIPseq	This paper	GEO: GSE296101
A549 Ad5-IκBαΔN ChIPseq	This paper	GEO: GSE296097

**Experimental models: Cell lines**		

Human: A549 lung adenocarninoma cell line (type II epithelial cells)	ATCC	CCL-185; RRID: CVCL_0023
Human: A549 2×GRE reporter cells	Chivers et al.^32^	NA
Human: A549 6κbtk reporter cells	Bergmann et al.^82^	NA

**Oligonucleotides**		

*CXCL8* qPCR primer; F: GCAGCTCTGTGTGAAGGTGC; R: AAAGGTTTGGAGTATGTCTTTATGCA	King et al.^30^	NA
*ICAM1* qPCR primer; F: TGCCCTGATGGGCAGTCAACA; R: GCAGCGTAGGGTAAGGTTCTTG	King et al.^30^	NA
*GAPDH* qPCR primer; F: TTCACCACCATGGAGAAGGC; R: AGGAGGCATTGCTGATGATCT	King et al.^30^	NA
*CXCL8* ChIP-PCR primer; F: GAGCACTCCATAAGGCACAA; R: TTCCTTCCGGTGGTTTCTTC	Altonsy et al.^10^	NA
*FKBP5* ChIP-PCR primer; F: TAACCACATCAAGCAAGCTG; R: GCATGGTTTAGGGGTTCTTG	Altonsy et al.^10^	NA
*KLF4* ChIP-PCR primer; F: ACTATGAAGCAACCAGGGAAG; R: CTTCCAGAACATTTGGTCCTTTG	This paper	NA
*EFNA1* ChIP-PCR primer; F: CAAAGGAGTCGCAATCCAGTA; R: GAAATTCCTCTGAGGTCTTGGG	This paper	NA
*MYOD1* ChIP-PCR primer; F: TGCAGGAGATGAAATACTAAGCAAGTA; R: AGATTGGAAACTGAGGACTTTAGTTAGAG	Altonsy et al.^83^	NA
*MYOG* ChIP-PCR primer; F: CCAATGAGACTGAGTGGGTTTTC; R: TCACCAGAGAAGACTGCTTTGC	This paper	NA
*OLIG3* ChIP-PCR primer; F: GGCAAGGACAGAGACAATCATA; R: CTCTGTGTTCTCGCTTTGGA	Altonsy et al.^83^	NA

**Software and algorithms**		

R (v4.2.2)	R Project	https://www.r-project.org
ImageLab (v6.0.1)	BioRad	https://www.bio-rad.com
Fiji ImageJ (v2.9.0/1.53t)	Schindelin et al.^84^	https://imagej.net/software/fiji/
Huygens software (v21.10.1)	Scientific Volume Imaging	https://svi.nl/
DiAna ImageJ (v1.48)	Gilles et al.^85^	https://imagej.net/plugins/distance-analysis
Bowtie2 (v2.4.4)	Langmead et al.^86^	https://github.com/BenLangmead/bowtie2
samtools (v1.18)	Danecek et al.^87^	https://github.com/samtools/samtools
MACS2 (v2.2.7.1)	Zhang et al.^88^	https://github.com/macs3-project/MACS/
diffBind (v3.2)	Ross-Innes et al.^89^	https://bioconductor.org/packages/DiffBind/
MEME-ChIP (v5.3.3)	Machanick et al.^90^	https://meme-suite.org/meme/tools/meme-chip
deepTools (v3.5.1)	Ramirez et al.^91^	https://github.com/deeptools/deepTools/
Gviz (v1.42.1)	Hahne and Ivanek.^92^	https://github.com/ivanek/Gviz
Kallisto (v0.48.0)	Bray et al.^93^	https://github.com/pachterlab/kallisto
Sleuth (v0.30.1)	Pimentel et al.^94^	https://github.com/pachterlab/sleuth
bedtools v2.31.1	Quinlan & Hall.^95^	https://github.com/arq5x/bedtools2/releases/tag/v2.31.1

**Other**		

JASPAR CORE database	Mathelier et al.^96^	https://jaspar.elixir.no
Database for Annotation, Visualization, and Integrated Discovery	Huang et al.^97^	https://davidbioinformatics.nih.gov/

### Experimental model and study participant details

#### Cell culture and stimuli

The human pulmonary type II cell line, A549, was obtained from ATCC (but not otherwise authenticated) and was grown under standard culture condition using Dulbecco’s modified Eagle’s medium (DMEM) supplemented with 10% fetal bovine serum (FBS), plus 2 mM L-glutamine. Cells were periodically tested for mycoplamsa infection, but in all instances tested negative. A549 cells containing the NF-κB reporter, 6κBtk.luc, and the GRE reporter, 2×GRE.luc, as previously described,^32^^,^^82^ were grown in growth media, as above, supplemented with 0.6 mg/ml G-418. Cells were incubated at 37°C in 5% CO_2_ and were grown to confluence either in 100 mm, or 6/12/24-well plates, as appropriate. Prior to experiments, cells were incubated overnight in basal media that was serum- and additive-free. Budesonide was dissolved in DMSO as a 10 mM stock. Unless specifically indicated, budesonide was used at 300 nM on cells, representing a concentration that was maximally effective/near maximal on a 2×GRE reporter and gene expression in A549 cells.^32^^,^^41^^,^^98^ Final DMSO concentrations on cells were ≤0.1%. Recombinant human IL1B was dissolved in phosphate-buffered saline (PBS) containing 0.1% bovine serum albumin (BSA). IL1B was previously found to be maximally or near maximally effective on NF-κB reporters and gene expression at 1 ng/ml and this concentration was used in all experiments unless specifically indicated.^35^^,^^67^

### Method details

#### Protein extraction and immunoblotting

Cells were lysed in 1× RIPA buffer (Cell Signaling) containing phosphatase and protease inhibitors prior to sonication for 10 min at 4 °C. Lysates were then mixed 1:3 with 4× Laemmli sample buffer (4% SDS, 10% 2-mercaptoehtanol, 20% glycerol, 0.004% bromophenol blue, 0.125 M Tris HCl, pH 6.8.), boiled for 10 min and loaded onto polyacrylamide gels.

For cytoplasmic-nuclear fractionation, cells were washed with ice-cold Hank’s balanced salt solution (HBSS) prior to scraping in 500 μL ice-cold HBSS supplemented with protease inhibitor cocktail (PIC) (Thermo). The cell suspension was transferred into 1.5 mL microcentrifuge tubes and centrifuged at 5,000*g* for 5 min at 4°C. Cell pellets were resuspended in 150 μL of ice-cold Cytoplasmic Extraction Reagent (CER) I (Thermo) supplemented with PIC prior to vortexing vigorously for 15 s to resuspend the pellet. Cell suspensions were incubated on ice for 10 min before 8.25 μL of ice-cold CER II (Thermo) was added to the tube, mixed by vortexing for 5 s, and incubated on ice for 1 min. Tubes were then centrifuged at 16,000*g* for 5 min at 4°C. Supernatants (cytoplasmic fraction) were transferred to pre-chilled 1.5 mL microcentrifuge tubes and the pellets were washed in ice-cold HBSS plus PIC. After centrifugation (16,000*g*, 5 min, 4°C), pellets (nuclear fraction) were lysed in 150 μL 1×RIPA buffer and sonicated for 10 min at 4°C. Cytoplasmic and nuclear fractions were mixed 1:3 with 4×Laemmli sample buffer and boiling for 10 min before loading onto polyacrylamide gels. Size-fractionated proteins were electro-transferred to nitrocellulose membranes and probed with primary antibodies overnight at 4°C. After washing and incubation with horseradish peroxidase-linked secondary immunoglobulin (Jackson ImmunoResearch), immune complexes were visualized by enhanced chemiluminescence (BioRad) using a ChemiDoc Imaging System (BioRad) prior to analysis using ImageLab software (Bio-Rad). Band volumes for proteins of interest were normalized to GAPDH.

#### Luciferase reporter assays

A549 cells harboring the stably integrated NF-κB reporter, 6κBtk.*neo*,^82^ or the GRE reporter, pGL3.2×GRE.TATA.*neo*,^32^ were treated with cytokines and/or glucocorticoids. Cells were harvested after 6 h and luciferase assays were performed using the Firefly Luciferase Assay Kit 2.0 (30085, Biotium Inc.) using a 20/20n luminometer (Promega).

#### Immunofluorescence staining and imaging

A549 cells were grown on No. 1½ glass cover slips (Electron Microscopy Sciences) to ∼60% confluence before being serum/additive starved overnight. Following treatments for 1 h, cells were washed with PBS, fixed with 2% paraformaldehyde in PBS for 20 min at room temperature and then washed 3 times with PBS. Fixed cells were permeabilized using PBS plus 0.1% Triton X-100 (v/v) for 10 min. To reduce the non-specific signal from nuclei, cells were treated with Image-iT FX Signal Enhancer (Thermo# I36933) for 30 min before blocking in BlockAid (Thermo# B10710) for 1 h. Cells were incubated overnight at 4 °C with mouse monoclonal anti-GR (1:100) or rabbit monoclonal anti-RELA (1:100) diluted in BlockAid solution. After washing with PBS plus 0.1% Triton X-100 (v/v) 3 times, cells were incubated for 1 h with AF594 goat anti-rabbit, AF594 goat anti-mouse or STAR-RED goat anti-mouse diluted in BlockAid solution. Cover slips were washed with PBS plus 0.1% Triton X-100 (v/v) 3 times, rinsed with PBS, incubated in 0.1% PicoGreen (Invitrogen) for 1 h, before being washed with PBS and mounting on glass slides using ProLong Gold Antifade.

For overview confocal images, cells were stained with either mouse anti-GR or rabbit anti-RELA, each with their respective AF594 secondary antibody, or, for dual staining, both anti-GR and anti-RELA using STAR-RED and AF594 secondary antibodies, respectively. Slides were imaged on Nikon A1R+ laser-scanning confocal microscope. Single-stained slides were used to assess cellular distribution of each factor in response to treatment, while images from dual-stained slides were used only for visual representation. For simulated-emission depletion (STED) imaging, dual-stained (GR:STAR-RED and RELA:AF594) slides were imaged using an Aberrior STEDYCON microscope attached to a Nikon Ti Eclipse Widefield microscope and a 100×/1.45 objective. Images were deconvolved with Huygens software. Deconvolved images were analyzed using DiAna ImageJ plugin for the localization of STED signal and calculation of the distance (x, y) between spots in GR channel and nearest spot in RELA channel, and vice versa.^85^ As certain areas within the nucleus, likely nucleoli or possibly other structures, showed a marked paucity of GR and RELA spots, these regions were excluded such that distance analyses for nuclear factors were performed only within areas of dense GR or RELA localization. The average nearest-neighbor difference between the distance separating the centers of any 2 spots and their edges was calculated to be 69 nm and thus any pair of GR/RELA spots were considered touching (co-localized) when their centers were ≤69 nm apart, and reported as percentage of total number of spots. To evaluate how actual separation distance compared to that obtained by chance, 10 iterations of a random distribution of the same number of GR and RELA spots were generated and the distance between closest GR and RELA spots was assessed in each iteration. The actual and random data were compared by plotting each as a cumulative distribution of the distances of all spot pairs and by comparing the numbers of spot pairs that were ≤69 nm apart.

#### Proximity Ligation Assay (PLA)

A549 cells were grown on 8-well chamber slides to ∼60% confluency before being serum/additive starved overnight. Following treatments, cells were washed with warm PBS, fixed with warm 2% paraformaldehyde in PBS for 15 min at room temperature and then washed 3 times with PBS. Fixed cells were permeabilized using PBS plus 0.1% Triton X-100 (v/v) for 10 min. Proximity Ligation Assay was performed using rabbit monoclonal anti-RELA and mouse monoclonal anti-GR according to the manufacturer’s instructions for the Duolink *In-Situ* Red reagents (Sigma). Mouse monoclonal anti-IκBα and rabbit monoclonal anti-EP300 were used with RELA and GR antibodies, respectively, to examine the capacity to detect well-validated interactions. Slides were mounted using ProLong Gold Antifade with DAPI and imaged using a Quorum Diskovery Flex spinning disk microscope, where z stacks of spinning-disk confocal images were acquired with a 60×/1.4 objective and z-projected with maximum intensity using Fiji ImageJ Software for quantification. The number of particles in the red channel (PLA) were counted within or outside the detected nuclei (DAPI channel) prior to normalization to the area occupied by nuclei in the field.

#### Chromatin immunoprecipitation

A549 cells in 100 mm cell culture plates were grown to >80% confluence and serum/additive starved overnight before treatments. Protein-DNA cross-linking was performed by adding methanol-free formaldehyde to 1% in the culture medium and incubating for 10 min at room temperature. Formaldehyde was then quenched at room temperature for 5 min with 125 mM glycine prior to washing cells for 5 min with ice-cold PBS and scraping into ice-cold lysis buffer (5 mM PIPES pH 8.0, 1 mM EDTA, 85 mM KCl, 5% glycerol, 0.5% NP-40) supplemented with protease inhibitor cocktail (PIC). Cell suspensions were incubated at 4°C for 3 h with continuous agitation. Nuclei were collected by centrifugation (600 g, 5 min, 4°C) and resuspended in ice-cold nuclei lysis buffer (NLB) (1xPBS containing 1 mM EDTA, 5% glycerol, 0.5% sodium deoxycholate, 0.1% SDS, 1% NP-40) supplemented with PIC. Samples were sonicated at 4°C using a Bioruptor set for 28–30 high-power bursts with 30 s on-off cycles. Lysates were cleared by centrifugation (maximum speed for 15 min at 4°C) and supernatants used for immunoprecipitation. Protein G magnetic Dynabeads were preincubated with primary antibody (2.5μL/sample) overnight at 4 °C in NLB supplemented with PIC and 5 mg/mL BSA. After washing twice with ice-cold NLB plus PIC, the Dynabeads were incubated with cleared lysates overnight at 4°C in NLB supplemented with PIC and BSA. Beads were given 4 washes with ice-cold NLB containing 500 mM NaCl, followed by 4 washes with ice-cold LiCl buffer (20 mM Tris at pH 8.0, 1 mM EDTA, 250 mM LiCl, 0.5% NP-40, 0.5% sodium deoxycholate). Crosslinks were reversed by incubation in TE buffer (10 mM Tris-HCl,1 mM EDTA, pH 8.0) supplemented with 0.7% SDS and 0.2 mg/mL proteinase K for 3 h at 55 °C, then 16 h at 65°C. DNA was purified with a ChIP DNA Clean & Concentrator kit. ChIP-qPCR was performed using 2 μL of purified ChIP DNA using Fast SYBR Green Master Mix with a QuantStudio3 PCR system. Amplification conditions were: 95°C for 20 s then 40 cycles of 95°C for 3 s, 60°C for 30 s. ChIP-PCR data were normalized to the geometric mean of three negative control regions, OLIG3, MYOD1, and MYOG, not predicted to be occupied by GR or RELA.^99^

ChIP DNA samples as well as pooled input samples from 2 independent experiments were submitted to the Center for Health Genomics and Informatics, University of Calgary, for sequencing. Following library preparation (NEB Ultra II kit), samples were subjected to 100-cycle paired-end sequencing (2 × 50 bp) on Illumina NovaSeq 6000 using NovaSeq SP kit v1.5. Demultiplexing was performed using bcl2fastq conversion software (v2.18.0.12) and read quality was assessed using *FastQC* (v0.10.1). Good-quality reads were mapped to GRCh38 reference genome using bowtie2 (v2.4.4) and the resultant BAM files were sorted and indexed using *samtools* (v) prior to peak calling using *MACS2* (v2.2.7.1). MACS-identified peaks for GR and RELA were assessed by *DiffBind* R package (v3.2) to calculate differential binding in response to each treatment compared to no stimulation. Initial filtering and overlap functions were performed on all samples for both transcription factors such that reads were counted for the same 400 bp regions for both factors and with all treatments. Normalization and differential binding relative to no stimulation were then estimated for each factor separately. Binding to regions was considered significant where log_2_ normalized read counts (binding intensity) ≥4 log_2_ fold change when compared to no stimulation ≥1, and FDR ≤0.05. The effect of budesonide on IL1B-induced RELA recruitment, or IL1B on budesonide-induced GR recruitment, was calculated by subtracting log_2_(fold) for RELA-IL1B, or GR-budesonide, from that of IL1B-plus-budesonide for RELA or GR, respectively. Thus, regions where log_2_ GBR intensity for IL1B-plus-budesonide/budesonide was ≤−0.5 were defined as “decreasing”; between >−0.5 and <0.5, was defined as “unchanged”; and ≥0.5 was defined as “increasing”. The RBRs were grouped in a similar fashion when comparing RELA log_2_ fold change following IL1B-plus-budesonide to that of IL1B only. RNAP2 and Ad-IκBαΔN ChIP-seq experiments were assessed for the regions that significantly recruited GR or RELA in any treatment in the initial analysis. Due to the variability between the Ad-IκBαΔN ChIP samples in the number of reads that were successfully mapped to the reference genome, BAM files were normalized to account for such differences before the post-normalization “.bigwig” files were used for graphical representation in heatmaps and data summarization.

Genomic region heatmaps were generated using *plotHeatmap* function within *deepTools* (v3.5.1), while individual genomic regions showing the longest transcript for each gene locus were plotted using *Gviz* R package (v.1.42.1).

#### Motif analysis

Motif enrichment analysis of the GBRs and RBRs for each treatment was performed using MEME-ChIP suite.^42^ Regions of 400 bp centered on the summit of each peak was extracted from hg38 in FASTA format, uploaded to MEME-ChIP v5.3.3 (http://meme-suite.org/tools/meme-chip) and analyzed for central enrichment of known factor binding sites from HOCOMOCOv11-core-HUMAN database.^100^

To explore the distribution of strong GREs and NF-κB motifs with the behavior of GBRs and RBRs on IL1B-plus-budesonide co-treatment, JASPAR databases were searched for binding sites with score ≥400 for NR3C1 (MA0113.3) and NR3C2 (MA0727.1) (collectively considered GRE), and for REL (MA0101.1), RELA (MA0107.1), and RELB (MA1117.1) (collectively considered NF-κB). The occurrence of GRE or NF-κB motifs within each 400 bp GBR or RBR was used to categorize each region as containing GRE only, NF-κB only, GRE and NF-κB, or neither.

#### Adenovirus infection

As previously described,^38^ cells were grown to ∼70% confluency prior to infection with Ad5-GFP or Ad5-IκBαΔN adenovirus at the indicated MOIs in serum-containing medium. After 24 h, the cells were serum-starved prior to treatment. At MOI 30, >90% of A549 cells become infected and NF-κB is robustly inhibited by IκBαΔN over-expression.^38^ MOI 30 was therefore was used for experiments.

#### RNA extraction, qPCR and mRNA-sequencing

Total RNA was extracted using the NucleoSpin RNA Extraction kit (MN-740955, Macherey-Nagel) and cDNA prepared from 0.5 μg of RNA. After a 1:4 dilution, PCR was carried out on 2.5 μL of cDNA using Fast SYBR Green Master Mix (4385618, Thermo) with a QuantStudio3 PCR system (Thermo). Relative cDNA concentrations were obtained from standard curves generated by serial dilution of cDNA obtained glucocorticoid-treated samples analyzed at the same time as the experimental samples. Amplification conditions were: 95°C, 20 s; then 40 cycles of 95°C, 3 s with 60°C, 30 s. Primer pairs for regions/genes of interest are listed in the key resources table. Primers were designed using PrimerBLAST (NCBI) and were synthesized by the DNA synthesis lab, University of Calgary. Primer specificity was determined using dissociation (melt) curve analysis: 95°C, 3 s with 60°C for 30 s followed by ramping to 95°C at 0.1 °C/s with continuous fluorescence measurement. A single peak in the change of fluorescence with temperature indicated acceptable specificity of primers. Target quantity from 2 technical replicates was averaged and normalized to the mean quantity of GAPDH from the same cDNA sample.

RNA from A549 cells that were not stimulated or treated with IL1B, budesonide or the combination for 1, 2, 6, 12, and 24 h were submitted to the Center for Health Genomics and Informatics, University of Calgary, for sequencing. RNA sequencing libraries, from 4 independent experiments, were prepared using Illumina TruSeq Stranded mRNA Library Prep kits with the poly(A) mRNA magnetic isolation module as described by the manufacturer. Libraries were validated by D1000 Screen Tape assay on an Agilent 2200 TapeStation system and quantified using Kapa qPCR Library Quantification kits. Libraries were pooled and sequenced across 4 consecutive 75 cycle high-throughput sequencing kits on a NextSeq 500 instrument (Illumina, San Diego, CA) to generate ∼20 million reads per sample. Demultiplexing of the sequencing data was performed using bcl2fastq conversion software and read quality assessed sample using FastQC (Illumina). Good-quality reads were mapped to GRCh38/hg38 reference human transcriptome using kallisto with 100 bootstraps per sample.^93^ Normalization and differential expression analysis was performed using the R package, sleuth.^94^ Genes with low abundance (<5 estimated counts in at least 90% of all samples) were filtered out before subsequent analysis. The Wald test was used to determine fold change (b value) and false-discovery rate (FDR) for each treatment when compared to NS control at each time point separately. Differentially expressed genes (DEGs) were taken as showing FDR ≤0.05 and log_2_(fold) ≥1 or ≤ −1. The effect of budesonide on IL1B-induced genes, or IL1B on budesonide-induced genes, was calculated by subtracting log_2_(fold) for IL1B, or budesonide, from that of I + B. Thus, ≥1 signifies enhanced genes, ≤−1 reduced genes and <1/>−1 indicated genes that were then unchanged by co-treatment.

#### Mapping expressed genes to binding regions

All genes that were aligned to at least 5 reads in at least 5 of the 80 samples submitted for RNA-seq were deemed to be expressed. This identified 14,786 genes expressed in A549 cells. The TSS coordinates for the expressed genes were exported in a.BED file and the distance from each TSS to the nearest GBRs, RBRs, or random regions for each condition (each in a separate.BED file) was calculated using *closestBed* function in *bedtools v2.31.1*.^95^

#### Gene ontology analyses

Gene ontology (GO) analyses were performed using the functional annotation chart and clustering tools within the Database for Annotation, Visualization, and Integrated Discovery (DAVID; v2024q4).^97^ GBRs or RBRs, defined above (decreasing, unchanged or increasing), were mapped to the nearest human gene (human genome assembly GRCh38.p13) and the resultant genes lists were submitted to the DAVID using standard parameters (Tables S1A and S1B). The gene lists for increasing, unchanged and decreasing GRBs and RBRs were further filtered by the upregulated DEGs in the respective treatments (budesonide, or IL1B, for decreasing and unchanged GBRs, or RBRs; and, IL1B-plus-budesoinde for the increasing GBRs and RBRs) (Tables S2A and S2B). GO categories for extraction were limited to biological process (GOTERM_BP_DIRECT), molecular function (GOTERM_MF_DIRECT) and Kyoto Encyclopedia of genes and genomes (KEGG) pathway. Molecular function terms for protein binding and signal transduction were not depicted in visualizations as being too generic. The Benjamini correction for multiple testing of enrichment *p* values (*P*_*B*_) was used and *P*_*B*_ ≤ 0.05 was used to highlight enriched terms. For functional annotation clustering, the cluster enrichment scores are -log10 of the geometric mean of all enrichment *p* values within the cluster. As recommended,^97^ clusters with scores ≥1.3 (i.e., -log10(0.05)) were taken as indicating potential interest. Significant GO terms (Functional Annotation Chart output) and clusters with enrichment scores ≥1.3 are provided (Tables S1A, S1B, S2A, and S2B). In visualizations (Figures 3I, 3J, and 5I), the following abbreviations within GO terms were adopted: +ve, positive; -ve, negative; reg, regulation; RNAP2, RNA polymerase 2; trans^n^, transcription; lipopolysaccharide, LPS; HPV, herpesvirus infection.

### Quantification and statistical analysis

Data summarization figures were generated using R package “*ggplot2*”. Data, from *N* independent experiments, are summarized as line graphs depicting means ± standard error (SE) or as box-and-whiskers plots, where whiskers represent tenth and ninetieth percentile and boxes represent lower and upper quartiles and median values.

Statistical analysis was performed with R software. All comparisons in sequencing or STED randomization data were analyzed using Wilcoxon test, for pairwise comparison, or using Dunn’s test, for pairwise multiple comparisons of the data. In such cases, the numeric value of *p* value, or their log_10_ derivatives, were depicted on the figure if the *p* value ≤0.05. For comparing the means in datasets with fewer observations, Student’s *t* test was used when comparing 2 groups and one-way ANOVA with Tukey’s post hoc test was used when comparing more than 2 groups. In such cases, stars are used to indicate the range that *p* values fell in, where ∗, ∗∗, ∗∗∗, and ∗∗∗∗ represent *p* ≤ 0.05, *p* ≤ 0.01, *p* ≤ 0.001, and *p* ≤ 0.0001, respectively. Color coding in figures indicates the comparison groups.

## Supplemental information

Document S1. Figures S1–S19

Table S1A. Functional annotation analysis for GBRs and RBRs within 30 kb of all human genes: Functional annotation chart

Table S1B. Functional annotation analysis for GBRs and RBRs within 30 kb of all human genes: Functional annotation clustering

Table S2A. Functional annotation analysis for GBRs and RBRs within 30 kb of A549-expressed genes: Functional annotation chart

Table S2B. Functional annotation analysis for GBRs and RBRs within 30 kb of A549-expressed genes: Functional annotation clustering
