# Supplementary material for: NF-κB-dependent GR cistrome redistribution recruits GR to inflammatory genes but correlates with lesser glucocorticoid-mediated repression
Source: iScience. 2025 Nov 25;28(12):114206. doi: 10.1016/j.isci.2025.114206 (PMC12756578; doi:10.1016/j.isci.2025.114206)
Supplement: Document S1. Figures S1–S19 [file mmc1.pdf]

iScience, Volume *28*

Supplemental information

NF-κB-dependent GR cistrome redistribution recruits GR to inflammatory genes but correlates with lesser glucocorticoid-mediated repression

Mahmoud M. Mostafa, Amandah Necker-Brown, Alex Gao, Andrew J. Thorne, Akanksha Bansal, Lucy Swift, Annika M. Maj, Sarah K. Sasse, Pina Colarusso, Anthony N. Gerber, and Robert Newton

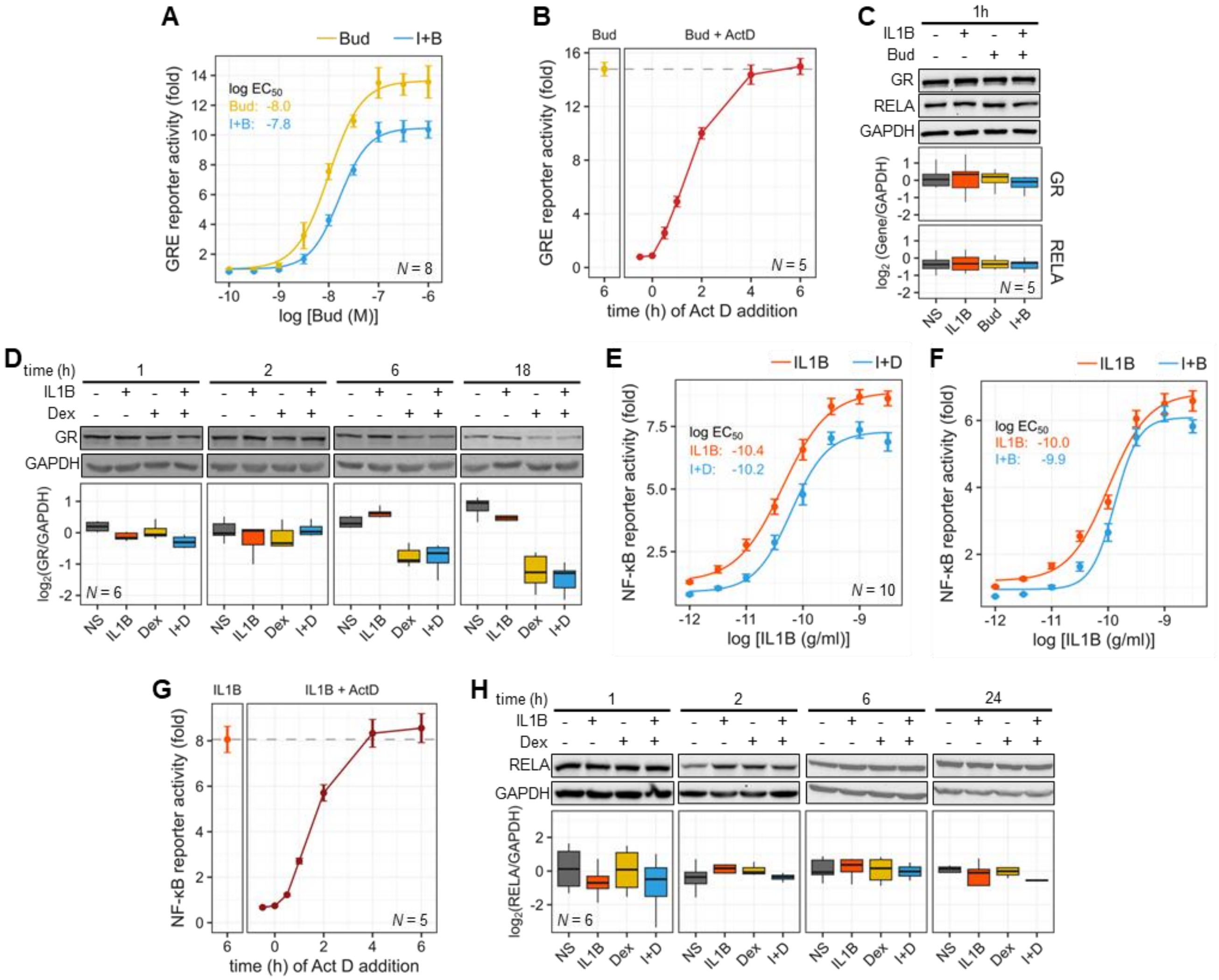

Figure S1. Effect of budesonide and/or IL1B on GRE- and NF-κB-reporter activity, time-dependence of GRE- and NF-κB-dependent transcription to inhibition by actinomycin D and effects of IL1B, budesonide and dexamethasone of GR and RELA expression.

(A) Budesonide-induced 2xGRE reporter (pGL3.2×GRE.TATA.neo) activity is modestly reduced by IL1B (1 ng/ml) co-treatment.

(B)Time-dependent inhibition by actinomycin D (10 μg/ml) of 2×GRE-dependent transcription when activated by budesonide (300 nM) for 6h.

(C) Neither IL1B (1 ng/ml) nor budesonide (300 nM) alone or combined affected GR or RELA expression at 1 h.

(D) Effect of dexamethasone (1 μM) and//or IL1B (1 ng/ml) for 1 – 24 h on GR expression.

(E) IL1B-induced NF-κB reporter (6κBtk.luc.neo) is modestly reduced by dexamethasone (1 μM) co-treatment (I+D).

(F) IL1B-induced NF-κB reporter (6κBtk.luc.neo) is modestly reduced by budesonide (300 nM) cotreatment (I+B).

(G)Time-dependent inhibition by actinomycin D (10 μg/ml) of NF-κB-dependent transcription when activated by IL1B (1 ng/ml) for 6 h.

(H) Effect of dexamethasone (1 μM) and//or IL1B (1 ng/ml) for 1 – 24 h on RELA expression.

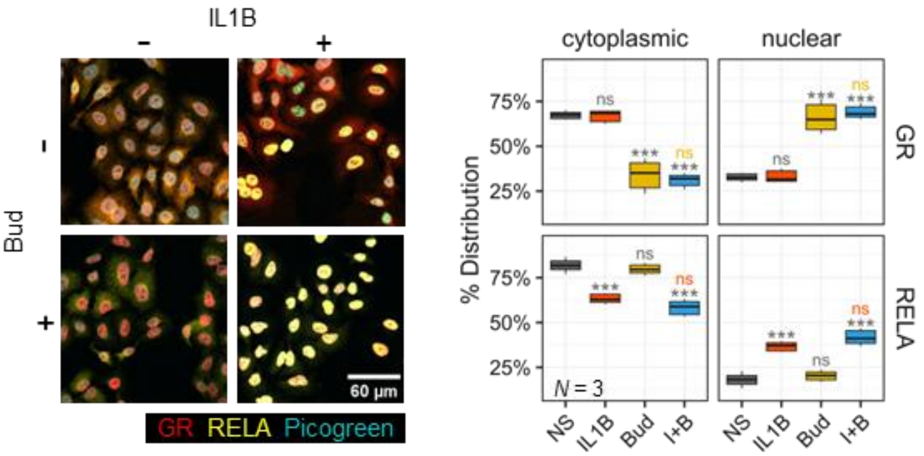

Figure S2. Nuclear translocation of GR and RELA following budesonide, or IL1B, respectively, was unaltered by co-treatment and led to nuclear co-localization of both factors.
Cells were either not stimulated (NS) or treated with IL1B (1 ng/ml) and/or budesonide (300 nM) for 1 h prior immunofluorescence detection of GR and RELA. The double-stand DNA dye, PicoGreen, was used to stain the nuclei. The fraction of each factor in the cytoplasm or nucleus, as a % of total, is plotted. Statistics were performed using ANOVA with Tukey post-test where grey is comparisons to NS, red is for comparisons to IL1B and gold indicates comparisons to budesonide. *** $P \leq 0.001$.

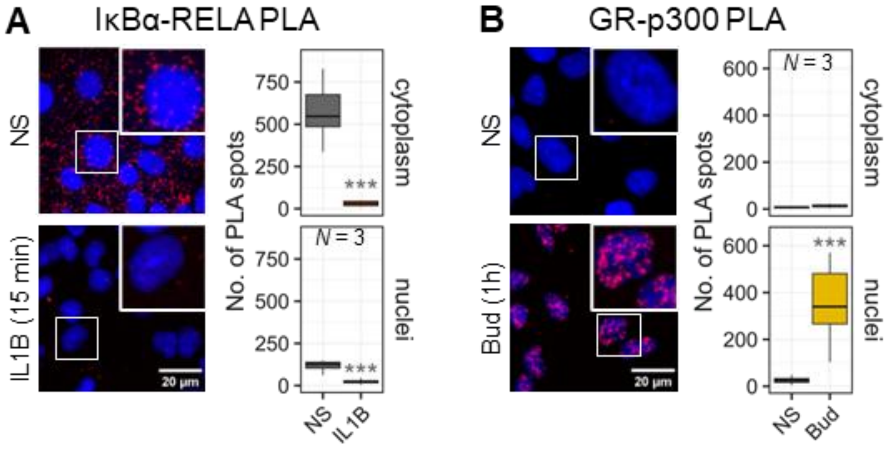

Figure S3. Proximity ligation assay (PLA) supports IL1B-dependent loss of cytoplasmic IκBα-RELA association, while budesonide promotes increased GR-p300 co-localization.
(A) Loss of IκBα-RELA interaction following 15 mins of IL1B treatment. Cells were not stimulated (NS) or treated with IL1B (1 ng/ml) for 15 mins prior to processing for PLA using anti-IκBα and anti-RELA antibodies.
(B) Budesonide promotes GR-p300 proximity. Cells were not stimulated or treated with budesonide (Bud, 300 nM) for 1 h prior to PLA using anti-GR and anti-p300 antibodies.
In both A & B, positive signal (number of PLA spots) are plotted for the cytoplasmic and nuclear compartments and are representative of *N* independent experiments. Significance compared to NS was tested using paired t-test. *** $P \leq 0.001$.

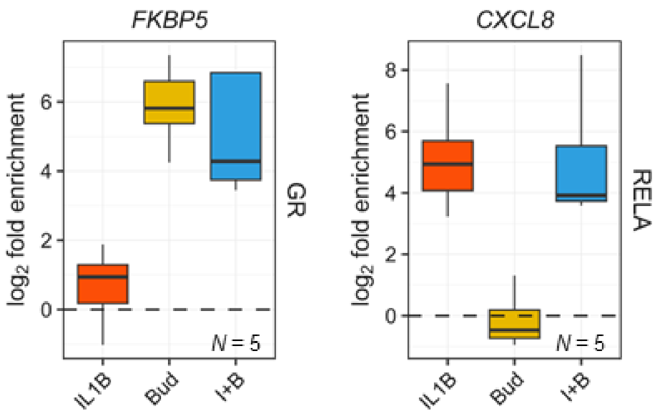

Figure S4. ChIP-PCR confirmed budesonide-induced enrichment of GR at the *FKBP5* locus and IL1B-induced RELA at the *CXCL8* locus.
Cells were either not stimulated or treated with IL1B (1 ng/ml) and/or budesonide (300 nM) for 1 h prior to fixing and processing for GR and RELA ChIP. ChIP-DNA from 5 independent experiments was subjected to qPCR using primers to detect a GBR in the 3' part of the *FKBP5* gene and a RBR just 5' to *CXCL8*. Robust budesonide-induced GR enrichment was detected at *FKBP5* and IL1B-induced RELA enrichment was shown at *CXCL8*.

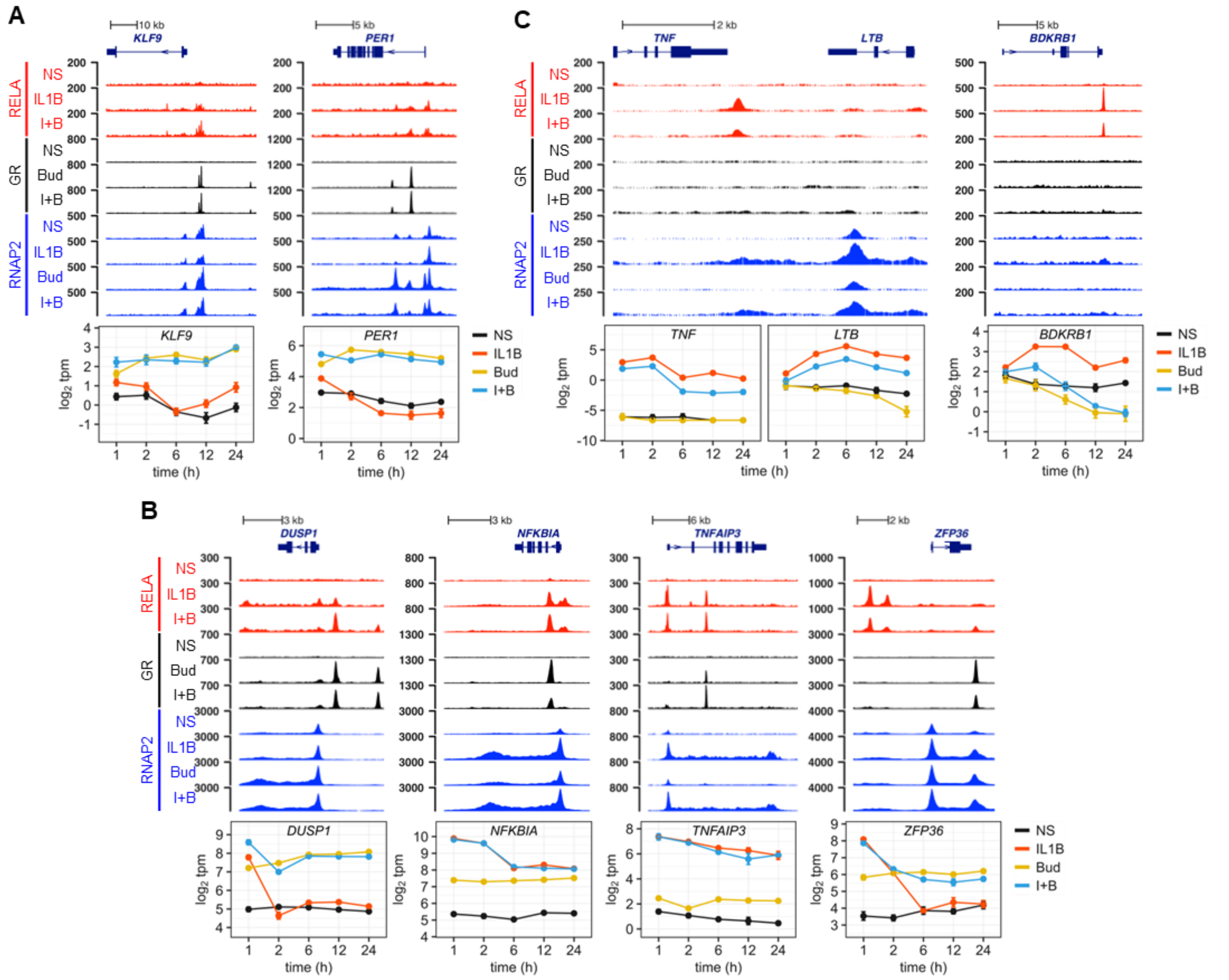

Figure S5. GR, RELA and RNAP2 recruitment to gene loci following 1h treatment with IL1B, budesonide or IL1B-plus-budesonide and effect on associated gene expression.
Cells were either not stimulated (NS) or treated with IL1B (1 ng/ml) and/or budesonide (300 nM) prior harvesting at 1 h for GR, RELA and RNAP2 ChIP-sequencing. Alternatively, cells were harvested after 1, 2, 6, 12 and 24 h and mRNA-sequencing (*N* = 4) was performed. GR, RELA and RNAP2 enrichment for selected loci are shown along with mRNA expression data (log_2 tpm) for the associated genes.
(A) Glucocorticoid-induced genes: *KLF9*, *PER1* are depicted to show primarily responsiveness to budesonide. Note: modest RELA recruitment at 1 h and IL1B-induced mRNA upregulation at some time points.
(B) Regulatory genes: *DUSP1*, *NFKBIA*, *TNFAIP3*, *ZFP36* are depicted which show independent GR and RELA recruitment as well as mRNA upregulation by budesonide and IL1B.
(C) IL1B-induced inflammatory genes: *LTB*/*TNF*, *BDKRB1* are depicted to show IL1B-induced recruitment of RELA that was reduced with IL1B-plus-budesonide. No evidence of GR recruitment was found and each gene revealed IL1B-upregulated mRNA expression that was reduced with IL1B-plus-budesoinde.

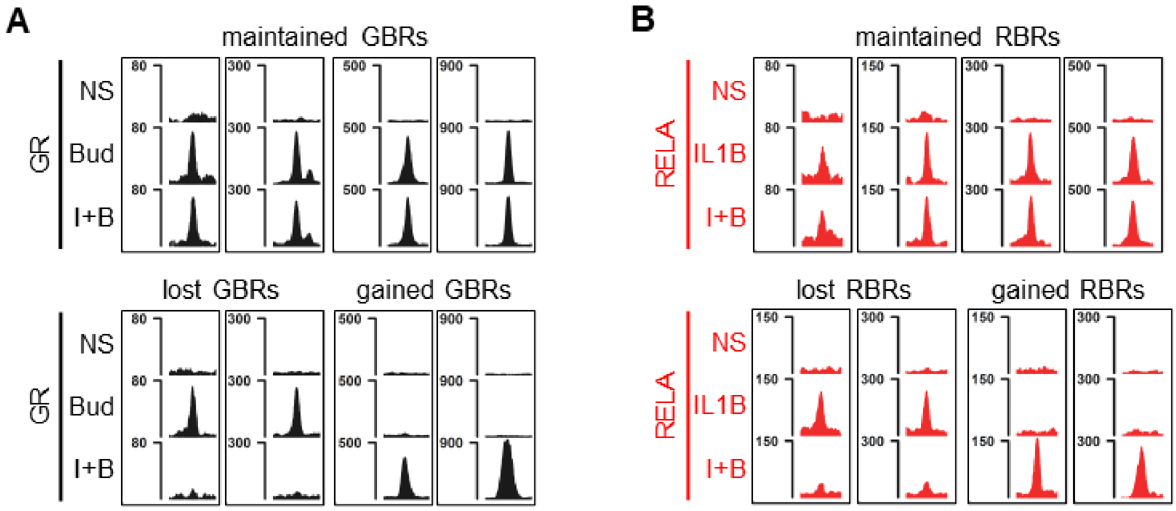

Figure S6. Loss, gain and maintenance of GBRs and RBRs are widely apparent on IL1B-plus-budesoinde treatment compared to each monotreatment.

(A) Budesonide-induced GBRs that were lost, gained or maintained with IL1B-plus-budesonide combination treatment compared to budesonide alone.

(B) IL1B-induced RBRs that were lost, gained and maintained with IL1B-plus-budesonide combination treatment compared to IL1B alone.

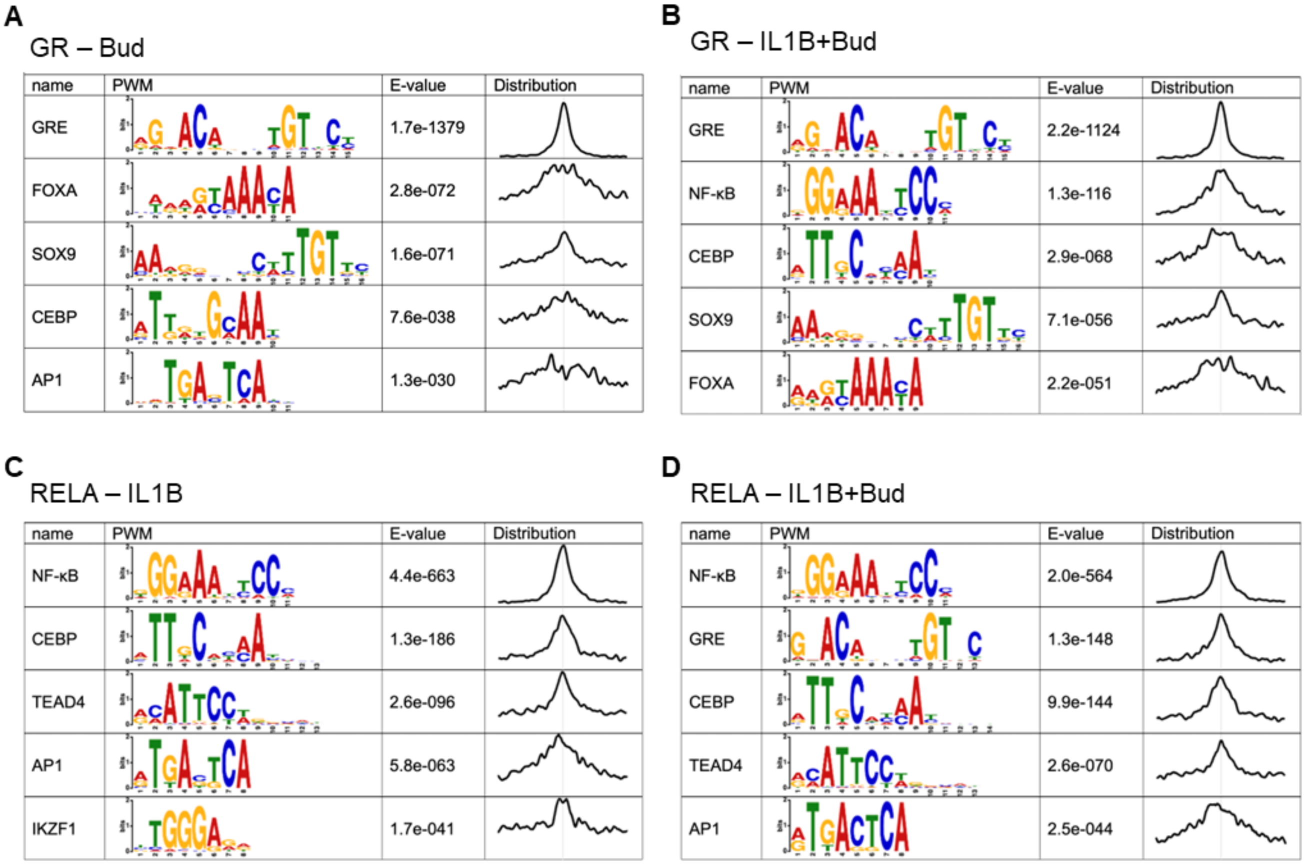

Figure S7. MEME-ChIP motif enrichment analysis of the GBRs induced by budesonide and IL1B-plus-budesoinde and the RBRs induced by IL1B and IL1B-plus-budesoinde.
(A) Top 5 most enriched transcription factor family motifs in the budesonide-induced GBRs.
(B) Top 5 most enriched transcription factor family motifs in the budesonide-plus-IL1B-induced GBRs.
(C) Top 5 most enriched transcription factor family motifs in the IL1B-induced RBRs.
(D) Top 5 most enriched transcription factor family motifs in the IL1B-plus-budesonide-induced RBRs.

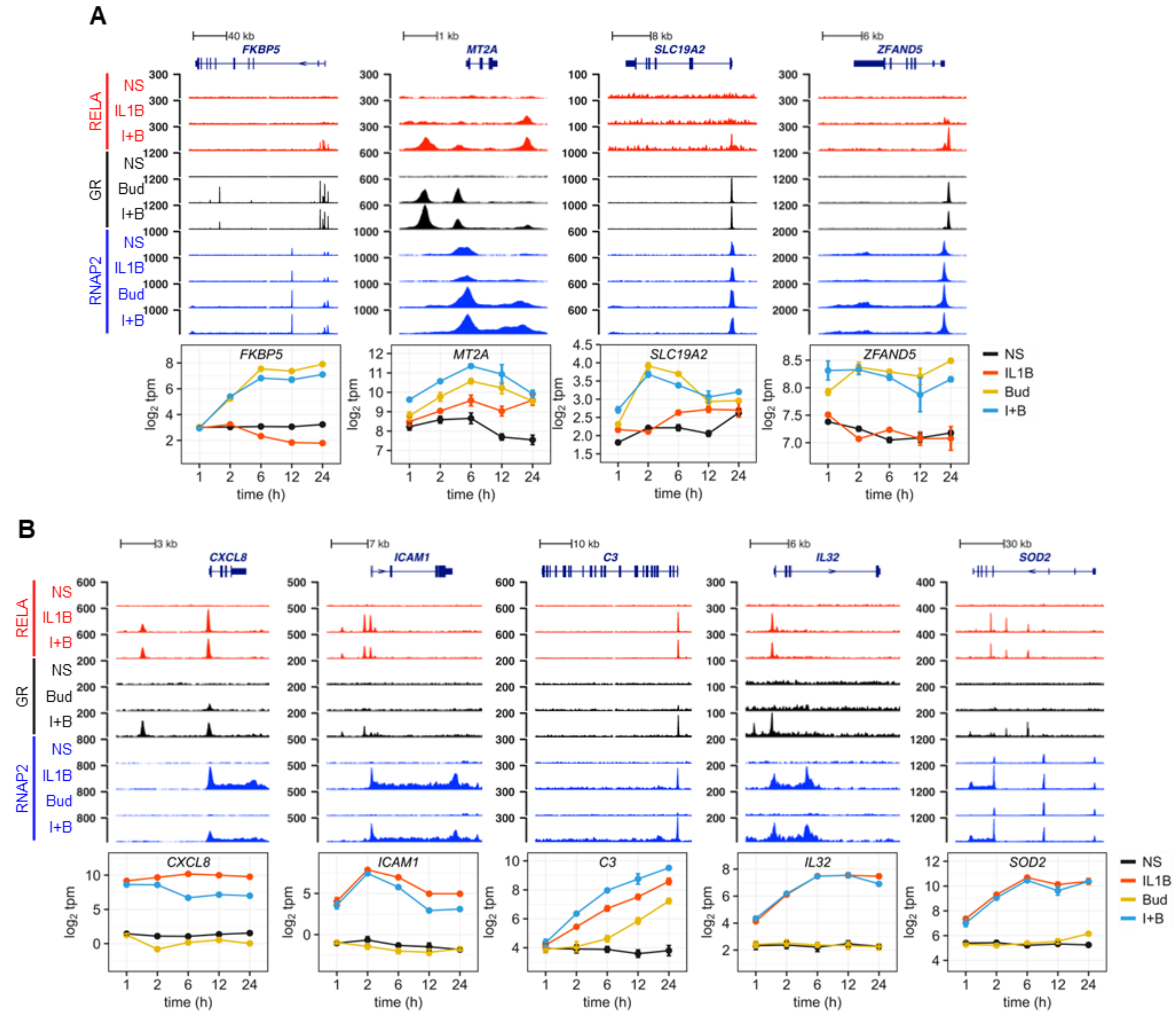

Figure S8. IL1B-plus-budesoinde co-treatment promotes the recruitment of RELA to budesonide-induced GBRs and the recruitment of GR to IL1B-induced RBRs.
Cells were either not stimulated (NS) or treated with IL1B (1 ng/ml) and/or budesonide (300 nM) prior harvesting at 1 h for GR, RELA and RNAP2 ChIP-sequencing. Alternatively, cells were harvested after 1, 2, 6, 12 and 24 h and mRNA-sequencing (N = 4) was performed. GR, RELA and RNAP2 enrichment for selected gene loci are shown along with mRNA expression data (log_2 tpm) for the respective genes.
(A) Glucocorticoid-induced genes: *FKBP5*, *MT2A*, *SLC19A2* and *ZFAND5* showing budesonide-induced GBRs that also recruit RELA on IL1B-plus-budesoinde co-treatment.
(B) IL1B-induced inflammatory genes: *CXCL8*, *ICAM1*, *C3*, *IL32*, and *SOD2* showing IL1B-induced RBRs that also recruit GR on IL1B-plus-budesoinde co-treatment.

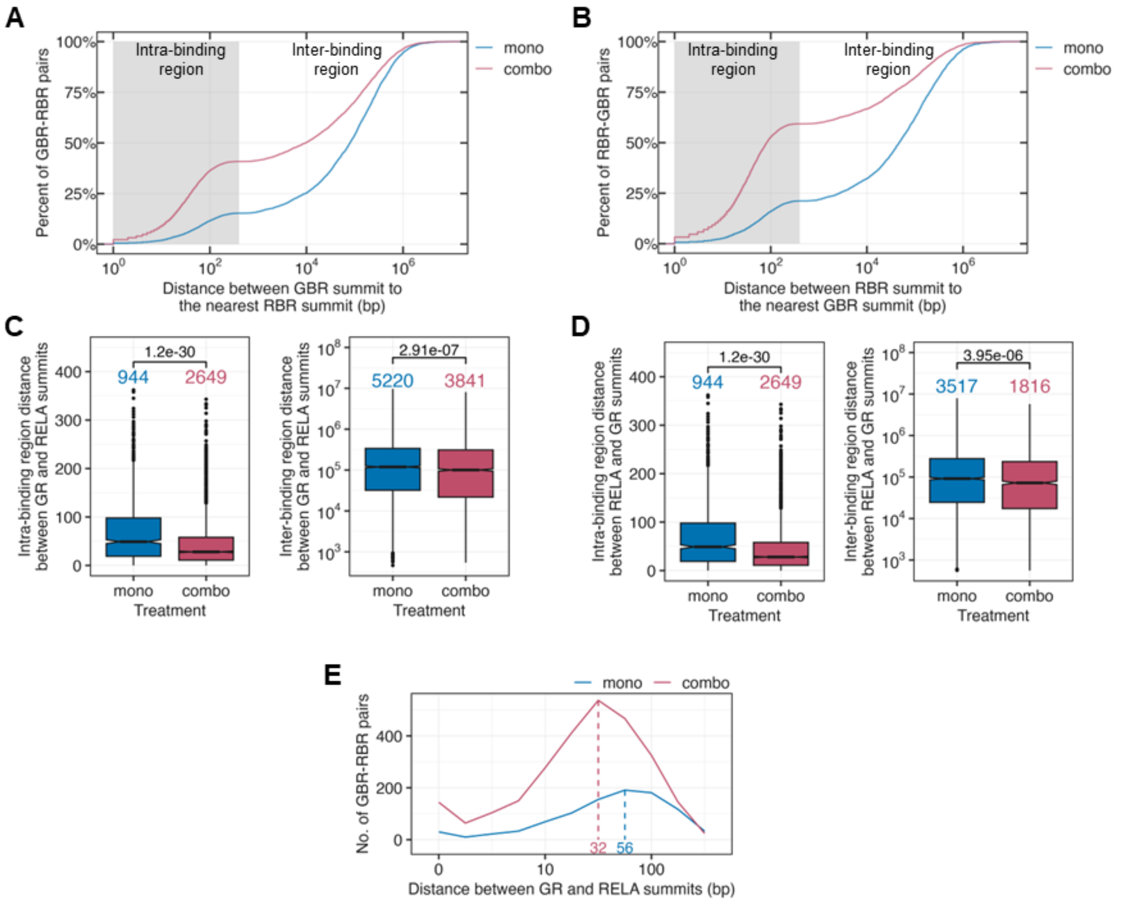

Figure S9. Distance between GBR and RBR summits in the IL1B and budesonide mono-treatments or in the IL1B-plus-budesonide cotreatment.

Using the data from figure 2 allowed identification of 6164 GBR and 4461 RBR summits induced by budesonide or IL1B, respectively. Likewise, IL1B-plus-budesonide (I+B) resulted in 6490 GBR and 4465 RBR summits. In panels A and B, grey shading (0 – 400 bp) highlights the summit-pairs broadly defined as being within the same binding region.

(A) Cumulative distribution of the distances between GBR summits to the nearest RBR summit in response to the mono-treatments (IL1B and budesonide; mono) or the I+B combination (combo) treatment.

(B) Cumulative distribution of the distances between RBR summits to the nearest GBR summit in response to the mono-treatments (IL1B and budesonide; mono) or the I+B combination (combo) treatment.

(C) Distances between GBR summits to the nearest RBR summit in response to mono- or combination treatments are plotted for summit-pairs located within the same 400 bp region (left) or across distinct (>400 bp) binding regions (right).

(D) Distances between RBR summits to the nearest GBR summit in response to mono- or combination treatments are plotted for summit-pairs located within the same 400 bp region (left) or across distinct (>400 bp) binding regions (right).

(E) Distribution of GBR-RBR summit distances in response to mono- or the combination treatments for summit-pairs located within the same 400 bp region (left panels from C & D). The modal average distance is indicated.

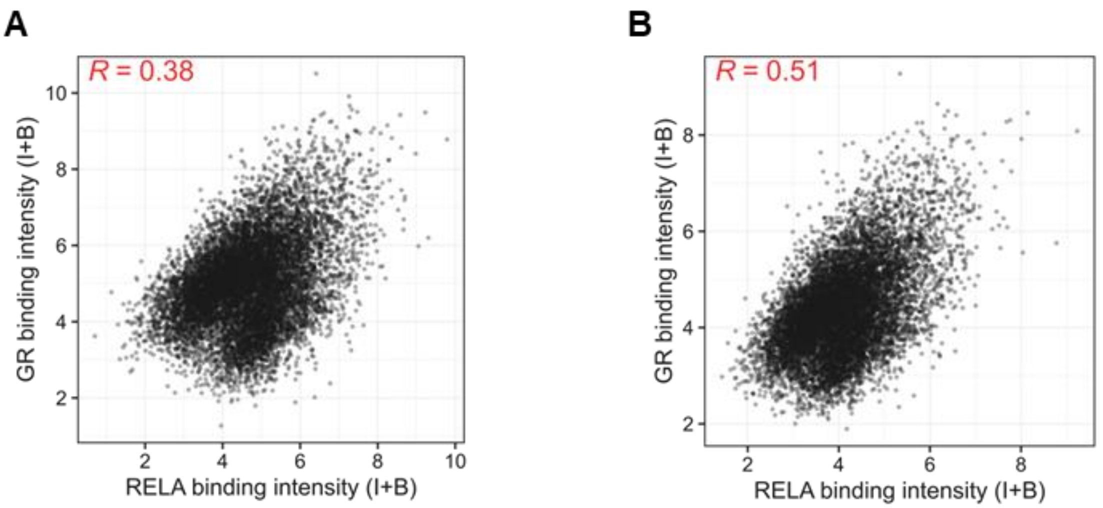

Figure S10. Positive correlation between GR and RELA binding intensity induced by IL1B-plus-budesoinde at all GBRs and RBRs. The GR and RELA binding intensity (log_2 normalized read count) for all the GBRs induced by budesonide and IL1B-plus-budesoinde (I+B) and all the RBRs induced by IL1B and I+B (total 10,740 regions) was plotted for each region.

(A) Positive correlation between RELA and GR binding intensity using 400 bp bins centered on each peak.

(B) Positive correlation between RELA and GR binding intensity using 1 kb bins centered on each peak.

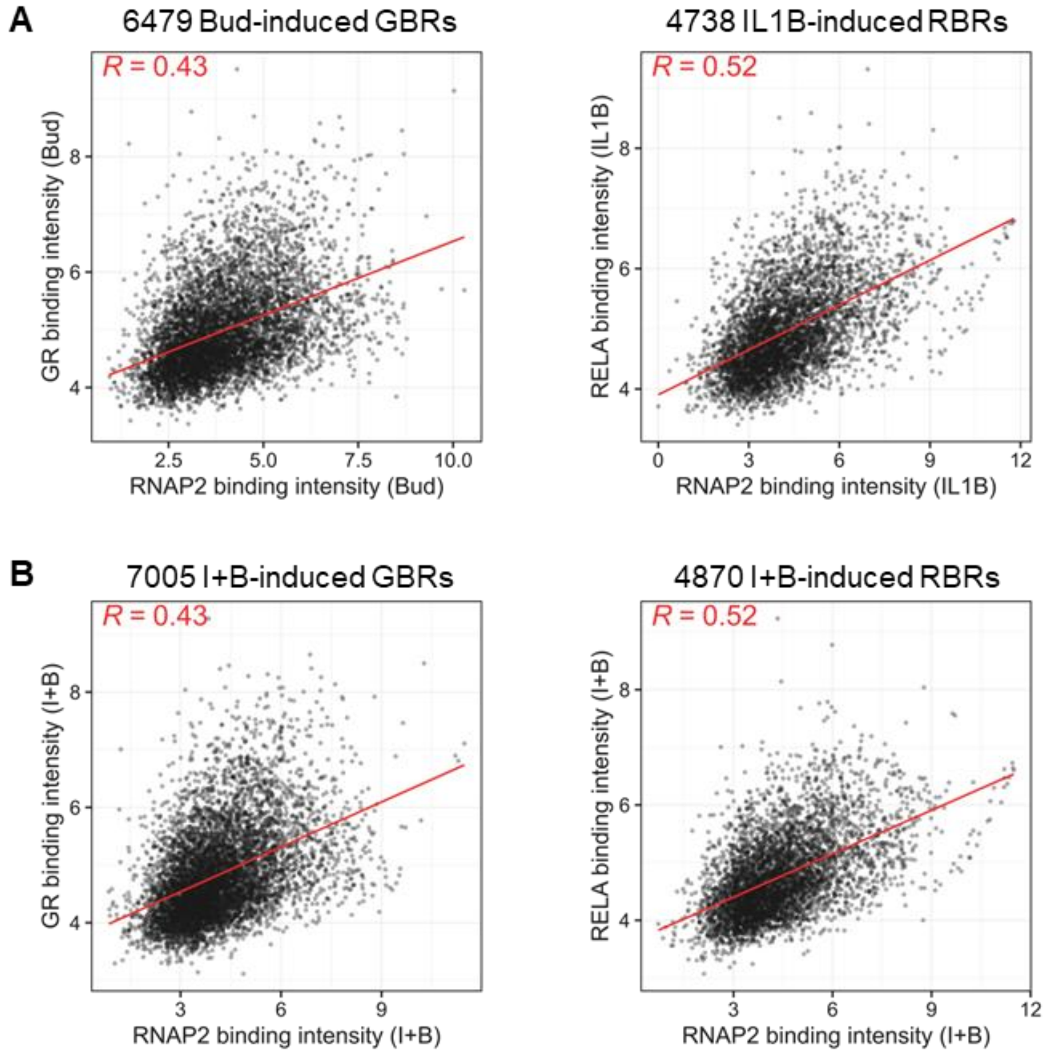

Figure S11. Enrichment of RELA at RBRs or GR at GBRs positively correlates with the presence of RNAP2. For each group of RBRs or GBRs the intensity of RELA or GR binding (as $\log_2$ normalized read count) was plotted against RNAP2 for each region.
(A) Intensity of budesonide-induced GBRs and IL1B-induced RBRs each positively correlates with the presence RNAP2.
(B) Presence of IL1B-plus-budesonide (I+B)-induced GBRs and RBRs each positively correlates with the presence RNAP2 in the cotreatment.

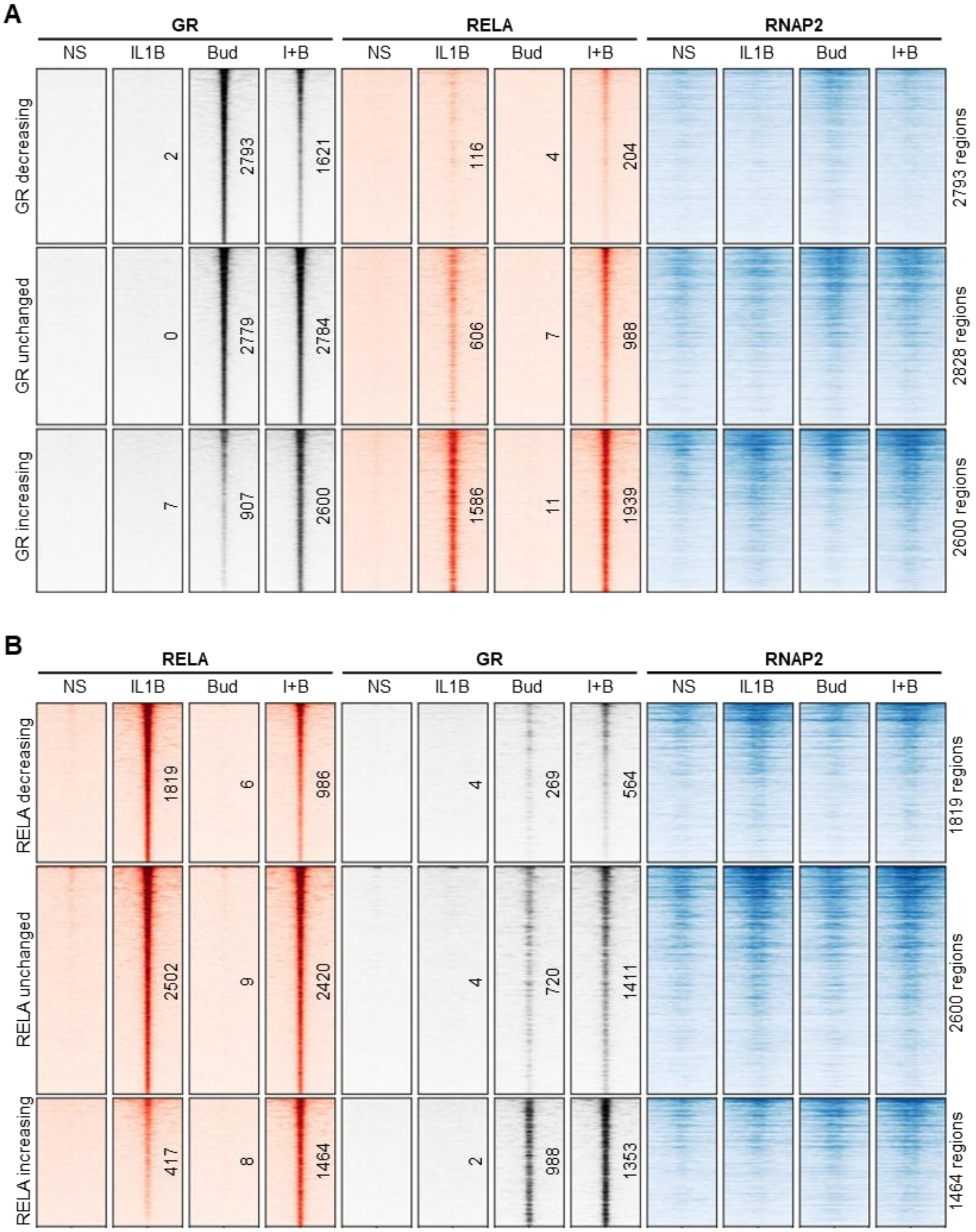

Figure S12. Binding of GR, RELA and RNAP2 at GBRs or RBRs ranked according to the change in GR, or RELA, binding on IL1B-plus-budesonide cotreatment respectively.
Cells were either not stimulated (NS) or treated with IL1B (1 ng/ml), budesonide (Bud, 300 nM) or IL1B-plus-budesonide (I+B) prior harvesting at 1 h for GR, RELA and RNAP2 ChIP-seq. Numbers on heatmaps indicate number of regions that meet the threshold criteria for binding and total number of regions in each group is provided at the far right.
(A) Heatmaps showing binding intensity for all 8221 GBRs separated and ranked as decreasing, unchanged and decreasing are shown along with their respective RELA and RNAP2 recruitment profiles.
(B) Heatmaps showing binding intensity for all 5883 RBRs separated and ranked as decreasing, unchanged and decreasing are shown along with their respective GR and RNAP2 recruitment profiles.

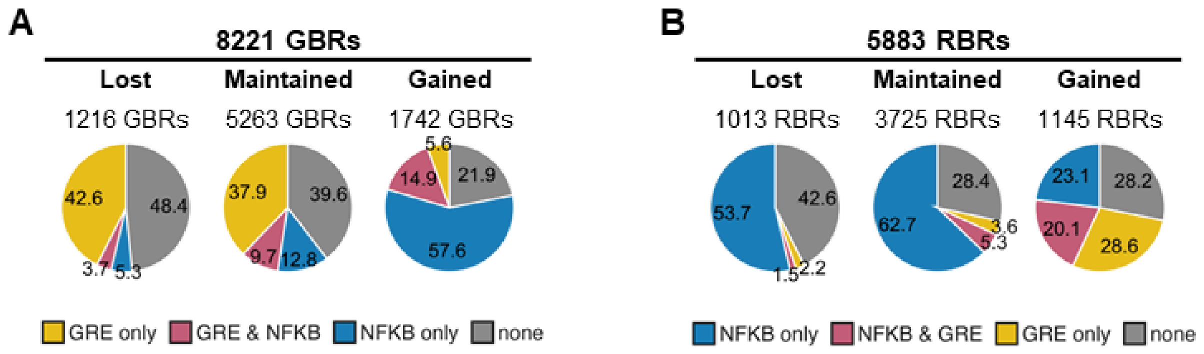

Figure S13. GRE and NF-κB motif distribution in lost, maintained and gained GBRs and RBRs. The 8221 GBRs and 5883 RBRs were grouped according to whether the binding region was lost, maintained or gained, as described in figure 3E. For each group, the presence of strong NF-κB and/or GRE motifs, as defined by JASPAR position-weight matrices with scores ≥400 (i.e. $P = 10^{-4}$), were plotted as pie charts.
(A) GRE and NF-κB motif distribution in lost, maintained and gained GBRs.
(B) NF-κB and GRE motif distribution lost, maintained and gained RBRs.

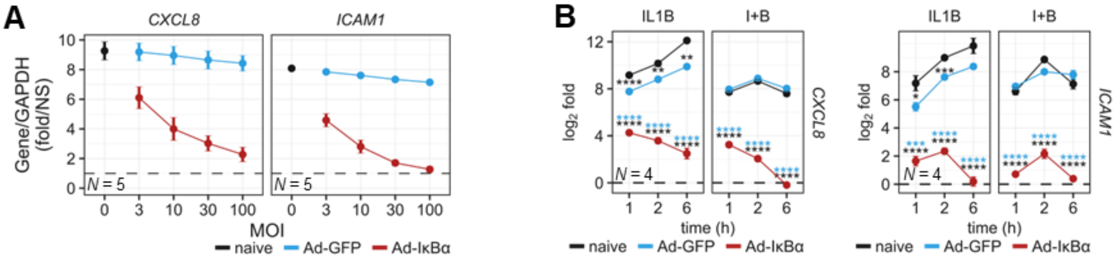

Figure S14. Effect of Ad5-IκBαΔN and Ad5-GFP on IL1B and IL1B-plus-budesonide-induced mRNA expression of CXCL8 and ICAM-1.

(A) Concentration-dependent inhibition of IL1B-induced CXCL8 and ICAM1 mRNA expression by Ad5-IκBαΔN, but not Ad5-GFP. Cells were either not infected (naïve) or infected with the indicated MOIs of each adenovirus prior to no stimulation or stimulation with IL1B (1 ng/ml). After 2 h, the cells were harvested for RNA and qPCR was performed for CXCL8, ICAM1 and GAPDH.

(B) At MOI 30, the effect of adenoviral over-expression of IκBαΔN, and GFP, on the expression of CXCL8 mRNA when induced by IL1B or IL1B-plus-budesonide is shown. Cells were either not infected (naïve) or infected with Ad5-GFP or AD5-IκBαΔN at MOI 30 prior to no stimulation or stimulation with IL1B (1 ng/ml) or IL1B-plus-budesonide (300 nM) (I+B). After 2 h, the cells were harvested for RNA and qPCR was performed for CXCL8, ICAM1 and GAPDH.

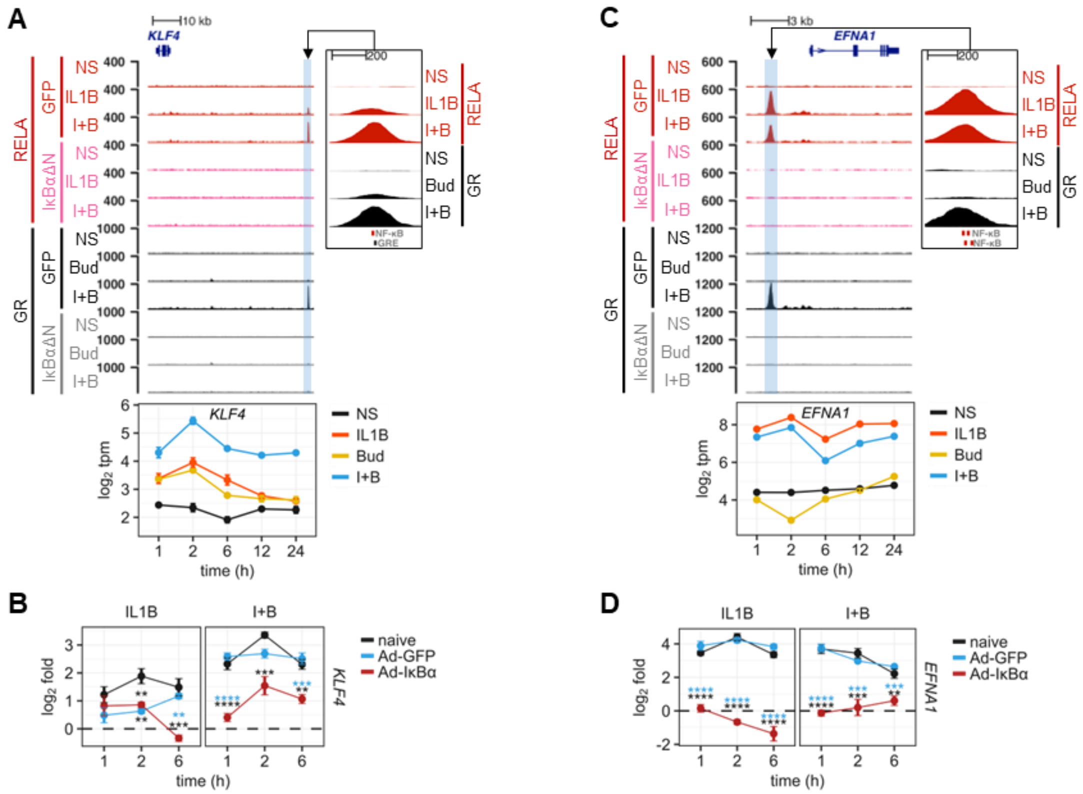

Figure S15. Increasing GBRs are located upstream of *KLF4* and *EFNA1*.
(A) *Upper right*, the increasing GBR/RBR located upstream of *KLF4* is shown is shown at high resolution using the RELA and GR ChIP-seq data from cells that were not stimulated (NS) or treated with IL1B (1 ng/ml), budesonide (300 nM) or IL1B-plus-budesoinde (I+B) for 1 h. The position of NF-κB and GRE motifs that meet the JASPAR score ≥400 are indicated. *Main figure*, Lower resolution RELA and GR ChIP-seq data are shown from cells that were infected with Ad5-GFP or AD5-IκBαΔN, each at 30 MOI, prior to treatments with IL1B, budesonide or IL1B-plus-budesonide for 1 h. *Below*, mRNA-seq data (*N* = 4) showing KLF4 mRNA expression expressed as $\log_2$ tpm following either no stimulation (NS) or treatment with IL1B, budesonide or IL1B-plus-budesoinde for the indicated times.
(B) Cells were either not infected (naïve), or infected with Ad5-GFP, or Ad5-IκBαΔN at MOI 30 prior to no stimulation or treatments with IL1B, budesonide or IL1B-plus-budesoinde. Cells were harvested for RNA at the indicated times and qPCR performed for KLF4 and GAPDH mRNA.
(C) *Upper right*, the increasing GBR located at the RBR upstream of *EFNA1* is shown is shown at high resolution using the RELA and GR ChIP-seq data from cells that were not stimulated (NS) or treated with IL1B (1 ng/ml), budesonide (300 nM) or IL1B-plus-budesoinde (I+B) for 1 h. The position of NF-κB motifs that meet the JASPAR score ≥400 are indicated. No GRE motifs were identified. *Main figure*, Lower resolution RELA and GR ChIP-seq data are shown from cells that were infected with Ad5-GFP or AD5-IκBαΔN, each at 30 MOI, prior to treatments with IL1B, budesonide or IL1B-plus-budesonide for 1 h. *Below*, mRNA-seq data (*N* = 4) showing EFNA1 mRNA expression expressed as $\log_2$ tpm following either no stimulation (NS) or treatment with IL1B, budesonide or IL1B-plus-budesonide for the indicated times.
(D) Cells were either not infected (naïve), or infected with Ad5-GFP, or Ad5-IκBαΔN at MOI 30 prior to no stimulation or treatments with IL1B, budesonide or IL1B-plus-budesonide. Cells were harvested for RNA at the indicated times and qPCR performed for EFNA1 and GAPDH mRNA.

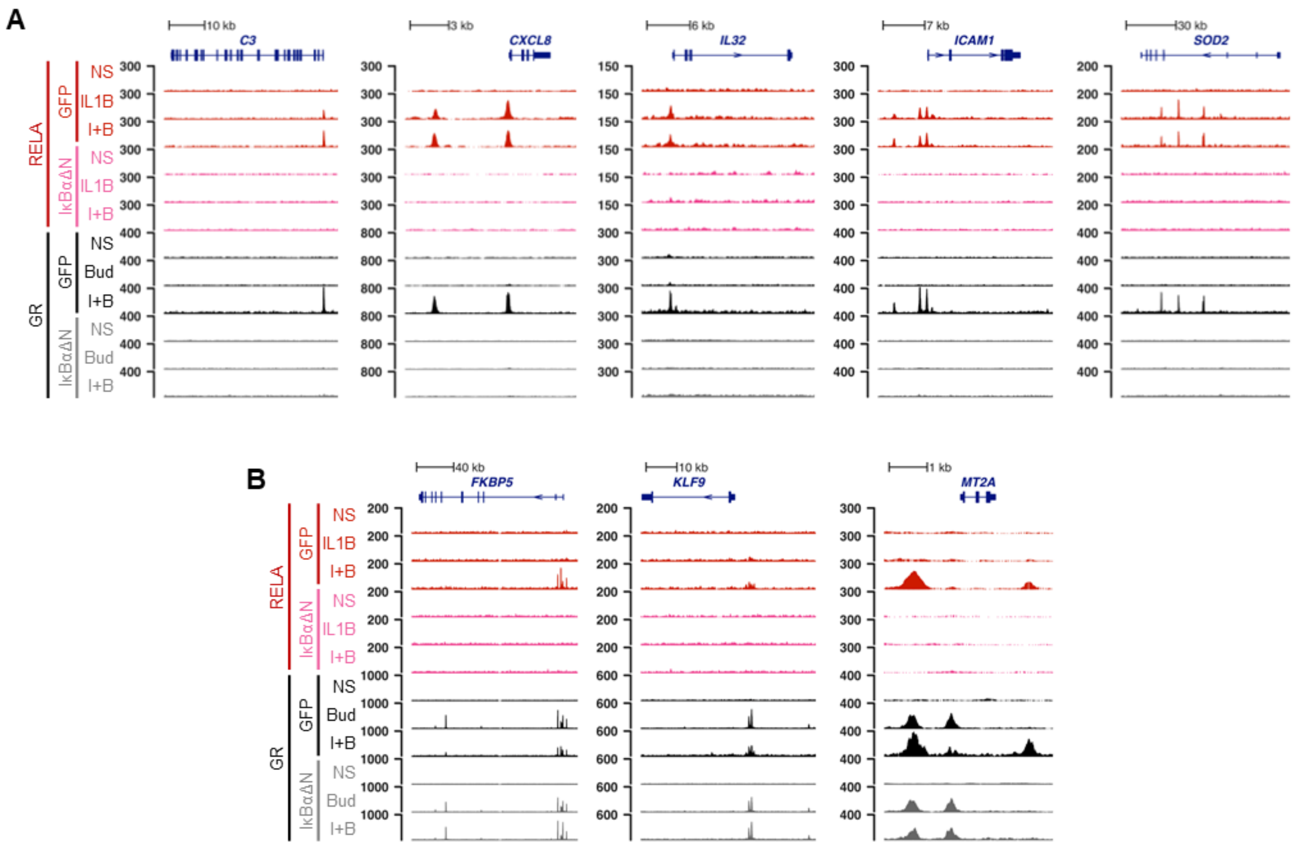

Figure S16. Recruitment of GR to RBRs in the presence of IL1B-plus-budesonide is blocked by IκBαΔN. RELA and GR ChIP-seq data are shown from cells that were infected with Ad5-GFP or Ad5-IκBαΔN, each at 30 MOI, prior to treatments with IL1B, budesonide or IL1B-plus-budesonide for 1 h.
(A) Inflammatory gene loci for: *C3*, *CXCL8*, *IL32*, *ICAM1* and *SOD2* show increasing GBRs that were prevented by IκBαΔN.
(B) Glucocorticoid-upregulated gene loci for: *FKBP5*, *KLF9* and *MT2A* show budesonide-induced GBRs that were unaffected by IκBαΔN. Note also how the reductions in the budesonide-induced GBRs by IL1B-plus-budesoinde for the 3′ GBR in *FKBP5* and the GBR just 3′ to *MT2A* appear to have been prevented by IκBαΔN.

A

IL1B

		Not induced	Induced	Totals
Bud	Not induced	13290 *(13141)*	1138 *(1287)*	14428
	Induced	731 *(880)*	235 *(86)*	966
	Totals	14021	1373	15394

logχ^2 p.val = -66.3

IL1B

	χ^2	Not repressed	Repressed	Totals
Bud	not repressed	12681 *(12306)*	1459 *(1834)*	14140
	Repressed	716 *(1091)*	538 *(163)*	1254
	Totals	13397	1997	15394

logχ^2 p.val = -236.2

B

IL1B

	χ^2	Not induced	Induced	Totals
Bud	not repressed	12958 *(12879)*	1182 *(1261)*	14140
	Repressed	1063 *(1142)*	191 *(112)*	1254
	Totals	14021	1373	15394

logχ^2 p.val = -15.4

IL1B

	χ^2	Not repressed	Repressed	Totals
Bud	Not induced	12660 *(12556)*	1768 *(1872)*	14428
	Induced	737 *(841)*	229 *(125)*	966
	Totals	13397	1997	15394

logχ^2 p.val = -23.7

Figure S17. Coregulation of gene expression by IL1B and budesonide is more than expected by chance. The DEGs up- or downregulated by IL1B or budesonide (Bud) at any time, as described in figure 5A and B, were subjected to Chi^2-testing.
(A) Chi^2-testing shows co-up- and co-downregulation by IL1B and budesonide to be more common than expected by chance.
(B) Chi^2-testing shows opposing regulation by IL1B and budesonide to be more than expected by chance.

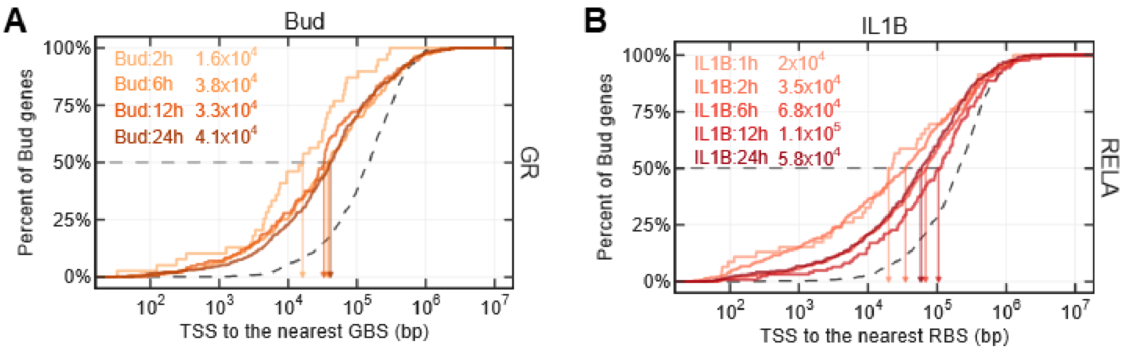

Figure S18. Relationship between timing of DEG upregulation and distance of TSS to nearest GR- or RELA binding region.

GBRs and RBRs induced by budesonide or IL1B, as shown in the ChIP-seq data from figure 2, were used to identify the closest binding region (GBR or RBR) for those DEGs showing peak mRNA upregulation by budesonide or IL1B, respectively, at 1, 2, 6, 12 or 24 h using the mRNA-seq data shown in figure 5A. TSS distance to equivalent numbers of random sites is shown (dotted line).

(A) Cumulative distribution of distances from TSS of budesonide (Bud)-upregulated DEGs at each time of peak expression to the nearest budesonide-induced GBR identified by GR ChIP.

(B) Cumulative distribution of distances from TSS of IL1B-upregulated DEGs at each time of peak expression to the nearest IL1B-induced RBR identified by RELA ChIP.

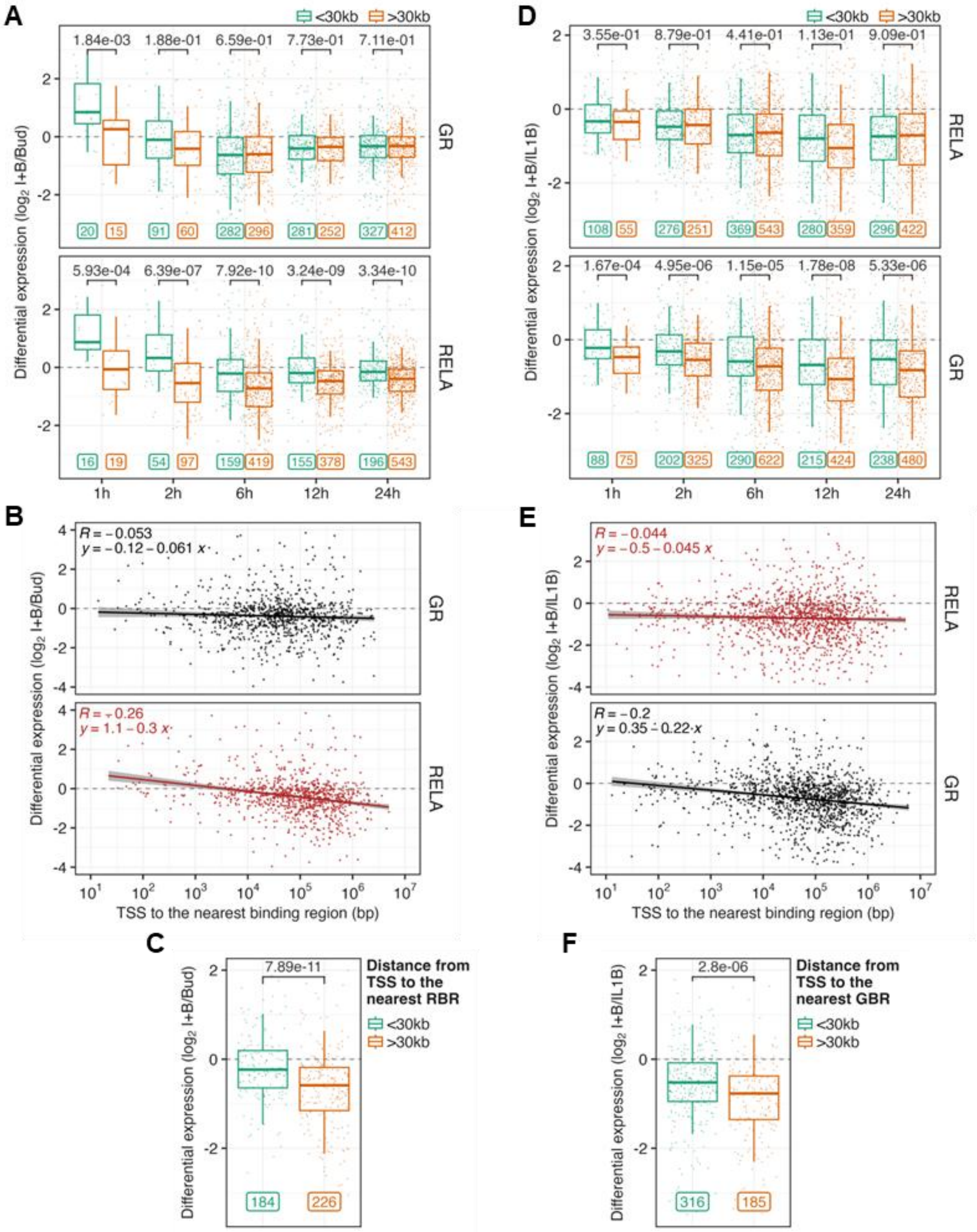

Figure S19. Correlation between binding region proximity and protection from expression loss in the IL1B-plus-budesonide (I+B) combination treatment.

(A) For all DEGs induced by budesonide (Bud) at each time, differential expression ($\log_2$(I+B/Bud)) is grouped by TSS proximity (≤/>30 kb) to the nearest GBR (Bud; top) or RBR (I+B; bottom).

(B) Differential expression ($\log_2$(I+B/Bud)) of Bud-induced DEGs at the time of peak induction plotted against distance from TSS to the nearest Bud-induced GBR (top) or I+B-induced RBR (bottom).

(C) For the 410 Bud-induced DEGs with TSS-GBR ≤30 kb (Fig. 6E), differential expression ($\log_2$(I+B/Bud)) at peak Bud-induction was grouped by TSS distance (≤/>30 kb) to nearest RBR (I+B).

(D) For all DEGs induced by IL1B at each time, differential expression ($\log_2$(I+B/IL1B)) is grouped by TSS proximity (≤/>30 kb) to the nearest RBR (IL1B; top) or a GBR (I+B; bottom).

(E) Differential expression ($\log_2$(I+B/Bud)) of IL1B-induced DEGs at the time of peak induction plotted against distance from TSS to the nearest IL1B-induced RBR (top) or I+B-induced RBR (bottom).

(F) For the 501 IL1B-induced DEGs with TSS-RBR ≤30 kb (Fig. 6I), differential expression ($\log_2$(I+B/IL1B)) at peak IL1B-induction was grouped by TSS distance (≤/>30kb) to nearest GBR (I+B).
